# Phylogenetic Analysis of Rare *Crepis* Species of the Crimean Flora (*C. purpurea* (Willd.) M. Bieb. and *C. callicephala* Juz.) Through Plastid Genome Reconstruction

**DOI:** 10.3390/ijms27219446

**Published:** 2026-10-23

**Authors:** Asan O. Emirsaliev, Dmitry A. Afonnikov, Irina V. Mitrofanova, Elena A. Salina, Alex V. Kochetov

**Affiliations:** 1 Institute of Cytology and Genetics, Siberian Branch of the Russian Academy of Sciences, 630090 Novosibirsk, Russia; 2 Kurchatov Center for Genome Research, Institute of Cytology and Genetics, Siberian Branch of the Russian Academy of Sciences, 630090 Novosibirsk, Russia; 3 Faculty of Natural Sciences, Novosibirsk State University, 630090 Novosibirsk, Russia; 4 N.V. Tsitsin Main Botanical Garden of the Russian Academy of Sciences, 127276 Moscow, Russia; 5 Institute of Fundamental and Applied Agrobiotechnologies, Siberian State University of Engineering and Biotechnology, 630039 Novosibirsk, Russia

**Keywords:** *Lagoseris*, plastome, chloroplast genome, *accD* pseudogenisation, acetyl-CoA carboxylase, Crepidinae, Cichorieae, Oxford Nanopore sequencing

## Abstract

One of the largest genera within the Cichorieae tribe of the Asteraceae family is *Crepis*, comprising ∼200 species. The diversity of numerous local forms is of interest for adaptation studies. There are two rare closely related species within the *Lagoseris* clade of the genus *Crepis* which occupy highly divergent habitats—*C. callicephala* Juz. and *C. purpurea* (Willd.) M. Bieb.—both occurring in the Crimean Peninsula. The habitats differ in environmental conditions and are isolated by location and altitude, restricting opportunities for gamete exchange. The species are similar morphologically but demonstrate different phenological patterns, representing a notable natural model for investigating how isolation and habitat conditions may shape genetic differentiation and local adaptation. We sequenced the total DNA of *C. callicephala* and *C. purpurea*. Relying on both short-read and long-read data, we assembled plastid genomes of these species and reconstructed the phylogeny of the subtribe Crepidinae. The assembled plastid genomes are 149,980 bp (*C. callicephala*) and 150,007 bp (*C. purpurea*), and the two species were recovered as a closely related pair on a long common branch within the sampled Crepidinae. We also noted an unusual structural rearrangement in the *rbcL*–*psaI* locus shared by the studied accessions of both species, affecting the *accD* gene (encoding the β-carboxyltransferase subunit of acetyl-CoA carboxylase), which has not been reported in other members of Cichorioideae. Together with the shifts in the inverted-repeat boundaries, these features provide molecular data relevant to the taxonomic status and relationship of *C. callicephala* and *C. purpurea*.

## 1. Introduction

Asteraceae is the largest family in the eudicot lineage, comprising nearly 1600 genera with 24,000–33,000 species spread throughout the world [1,2]. One of the largest tribes is Cichorieae possessing ~1550 well-supported species [3] and thousands of poorly defined species and local forms, contributed mainly by *Hieracium*, *Crepis* and *Taraxacum*. One of the largest genera within Cichorieae is *Crepis* belonging to the Crepidinae subtribe, with ~200 species being recognised [1]. The *Crepis* species occur in the Old and New World, spanning a wide range of environmental conditions across a broad geographical range. The diversity of numerous local forms might be of interest for adaptation studies. The genus *Crepis* comprises distinct evolutionary lineages, including *Crepis sensu stricto* and the *Lagoseris* lineage [4]. *Lagoseris* is also treated as a genus in regional floras and identification keys [5,6,7]. The Global Compositae Database lists it as an accepted genus, whereas Plants of the World Online places the name in synonymy with *Crepis* [8,9]. There are two rare closely related species within the *Lagoseris* clade which occupy highly divergent habitats—*C. callicephala* Juz. and *C. purpurea* (Willd.) M. Bieb.—both occurring in the Crimean Peninsula [10,11].

While the first species (*C. callicephala*) is confined to rock crevices at the front edge of the Main Ridge of the Crimean Mountains, with seeds also drifting onto colluvial taluses, at an altitude of 1000–1500 m above sea level [12], the second (*C. purpurea*) occurs on calcareous slopes in the foothills of the Outer and Inner Ridge at an altitude ranging between 200 and 450 m above sea level [5].

**Figure 1:**
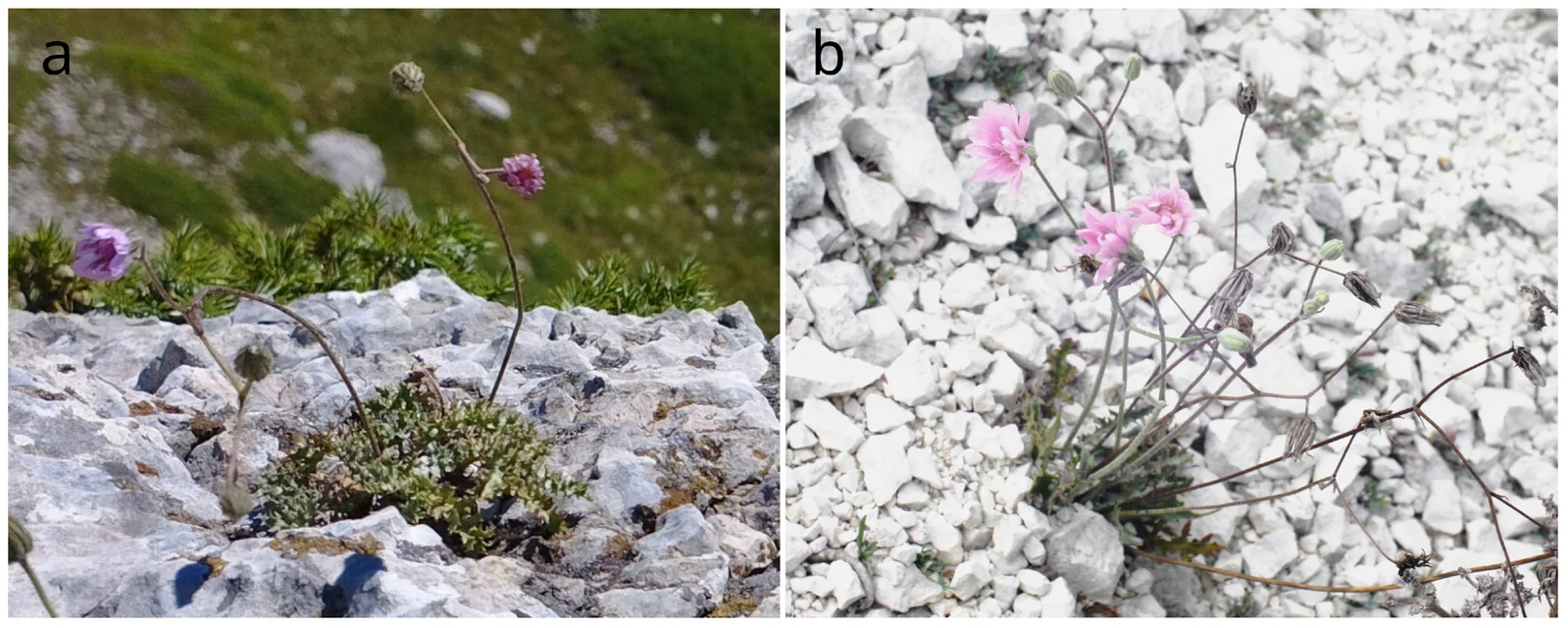
*Crepis callicephala* and *C. purpurea* in their natural habitats in Crimea. (**a**) *C. callicephala* on limestone near the summit of Eklizi-Burun (1527 m a.s.l.), Chatyr-Dag massif; (**b**) *C. purpurea* on calcareous rubble at the foot of Ak-Kaya, Crimean Foothills.

Although the two taxa are morphologically similar, traditional Crimean floristic treatments—in which these species are considered within the genus *Lagoseris*—separate them mainly by a combination of plant size, capitulum size, and the indumentum of the peduncles and involucral bracts. *Crepis callicephala* (Figure 1a) is generally described as the more robust taxon, with larger capitula and yellowish glandular hairs intermixed with bristles on the peduncles and involucral bracts, whereas *C. purpurea* (Figure 1b) is usually smaller, with a predominantly greyish, arachnoid pubescence on these structures. These distinguishing features are not consistent, however. Privalova’s *Lagoseris* account [5] notes specimens resembling *L. purpurea* but bearing glandular hairs on the peduncles and involucral bracts, and Abdulganieva [10] reported individuals with differing capitulum and peduncle pubescence in the Crimean populations examined.

The two taxa also differ in the seasonality of shoot development. In comparative observations, Nikiforov and Nikiforova [11] reported that a *C. callicephala* shoot completes its vegetative development over at least two growing seasons and becomes generative only after overwintering, whereas *C. purpurea* shoots can pass from the vegetative to the generative phase within a single warm season and, through the successive formation of such shoots, flower over a substantially longer period. This difference in flowering phenology, together with the geographical and altitudinal separation of the habitats, restricts opportunities for gamete exchange between the two.

Thus, these species represent a notable natural model for investigating how isolation and habitat conditions may shape genetic differentiation and local adaptation. Questions such as population fitness, local adaptations, the rate of gamete exchange and divergence time can be addressed within this system. As an initial step, we compare their complete plastid genomes and reconstruct plastome-based phylogenies to examine their relationship to each other and their placement among sampled Cichorieae, complementing earlier ITS- and *matK*-based studies of *Crepis* [13].

The taxonomic status of *C. callicephala* and *C. purpurea* remains challenging. Some taxonomic treatments place *C. callicephala* in synonymy with *C. purpurea* [14,15,16]. Other regional floras and identification keys recognise *L. callicephala* (≡*C. callicephala*) and *L. purpurea* (≡*C. purpurea*) separately [5,6,7]. The Global Compositae Database and *The Plant List* v1.1 also list *C. callicephala* as an accepted species [17,18]. The Red Data Books of the Republic of Crimea and Ukraine include *L. callicephala* within *C. purpurea* and *L. purpurea*, respectively [19,20]. The Ukrainian account also includes *L. robusta* [20]. The Sevastopol Red Data Book account uses *C. purpurea* but identifies its local populations as *L. purpurea s. str.* [21]. *Crepis purpurea* is also included in the *Red Data Book of Turkish Plants* [22] and assessed in the IUCN Red List [23]. Appendix I of the Bern Convention lists both generic combinations, *C. purpurea* and *L. purpurea* [24]. Material identified as *C. callicephala* and *C. purpurea* is maintained in the genebank of the Nikita Botanical Gardens (NBG) [25,26,27].

We followed the NBG genebank in recognising *C. callicephala* and *C. purpurea* as separate taxa and retained the original identifications of the studied material. Some authors of the mid-20th century also recognised one more species—*Lagoseris robusta* Czer. [5]. The diagnostic characters of *L. robusta*, *C. callicephala* and *C. purpurea* partly overlap, complicating their delimitation based on morphology alone.

We aimed to contribute to *Crepis* genomics by providing plastid genomes that are among the first reported for the clade *Lagoseris*. At the time of the original analysis (2023–2024), no complete *Crepis* plastome was available other than that deposited as *C. rigescens*.

## 2. Results

### 2.1. Sequencing, Plastid Genome Assembly and Variant Analysis of C. callicephala and C. purpurea

Total DNA of *Crepis callicephala* and *C. purpurea* was extracted from leaves of *in vitro* plantlets (Section 4.1). Eight sequencing runs of *C. callicephala* libraries on the Oxford Nanopore Technologies (ONT) MinION instrument yielded 8.3 Gb of long reads, and four runs of *C. purpurea* libraries produced 6.2 Gb. Illumina sequencing of *C. callicephala* libraries yielded 73.4 Gb of paired-end short reads with an insert size of 247 ± 50 bp.

**Figure 2:**
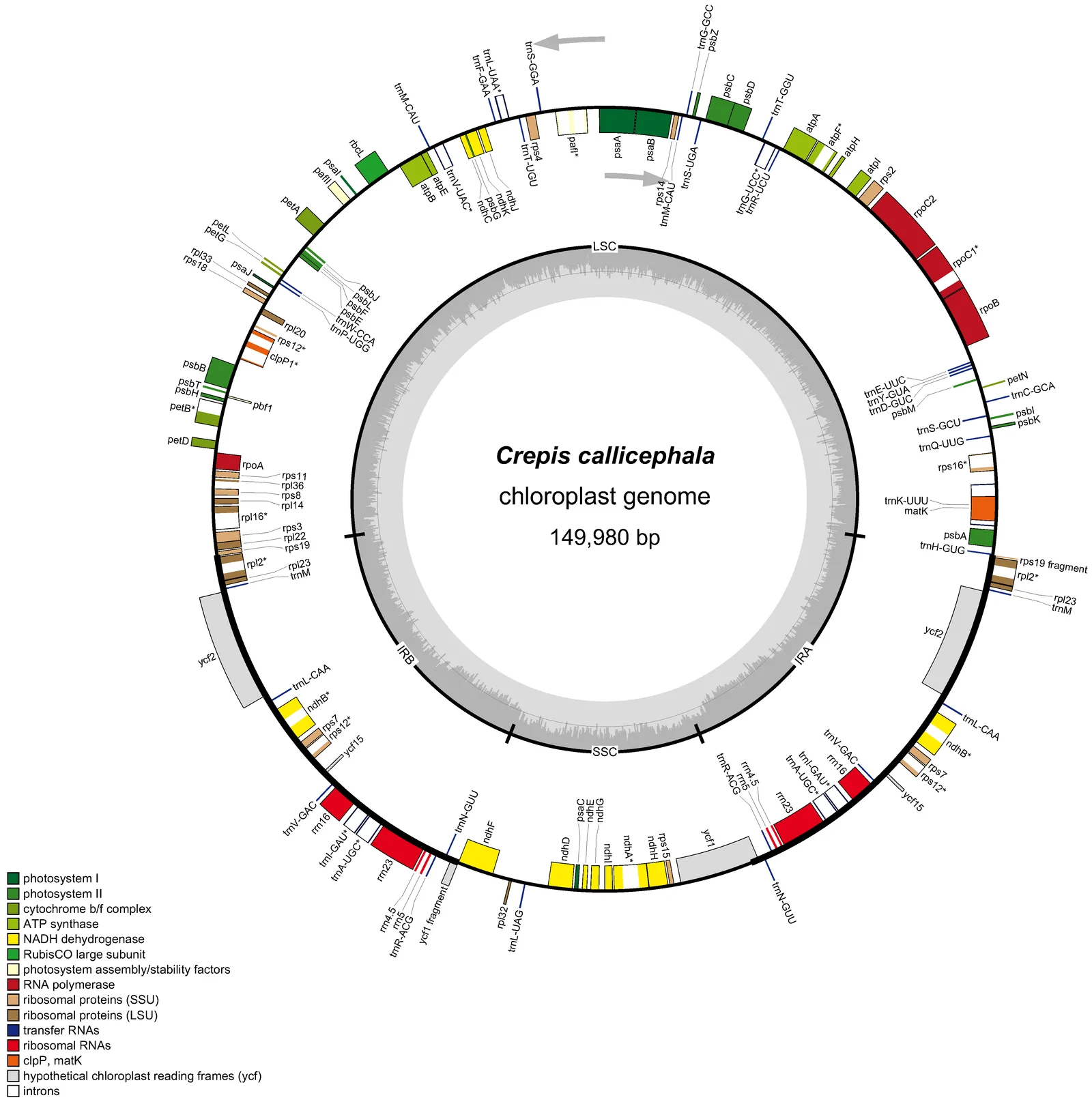
The map of the *Crepis callicephala* plastid genome. Colours indicate gene and feature categories as shown in the legend. Genes inside the circle are transcribed clockwise, whereas those outside are transcribed counterclockwise; grey arrows indicate the direction of transcription. Asterisks (*) mark intron-containing genes. The inner ring shows the local guanine–cytosine (GC) content as a dark-grey profile. LSC, large single-copy region; SSC, small single-copy region; IRA and IRB, inverted-repeat regions A and B. The disrupted *accD* locus (293 bp) between *rbcL* and *psaI* is not drawn.

Two plastid genome assemblies of *C. callicephala* were obtained: the first, with a length of 149,980 bp, was based solely on short-read data, and the second, with a length of 149,790 bp, resulted from the reference-guided assembly of the long reads. QUAST comparison showed that the long-read-derived assembly differed from the short-read assembly by 4 mismatches and 25 indels, all ≤5 bp, with no misassemblies detected (Table S1). Given the higher per-base accuracy of Illumina reads, the short-read assembly was retained as the primary sequence for subsequent analyses, whereas the long-read-derived assembly was used to assess concordance of the overall plastome structure. The visualisation plot of the short-read assembly annotation is presented in Figure 2. The plastid genome assembled possesses a typical quadripartite structure with two unique regions split by inverted repeats.

**Figure 3:**
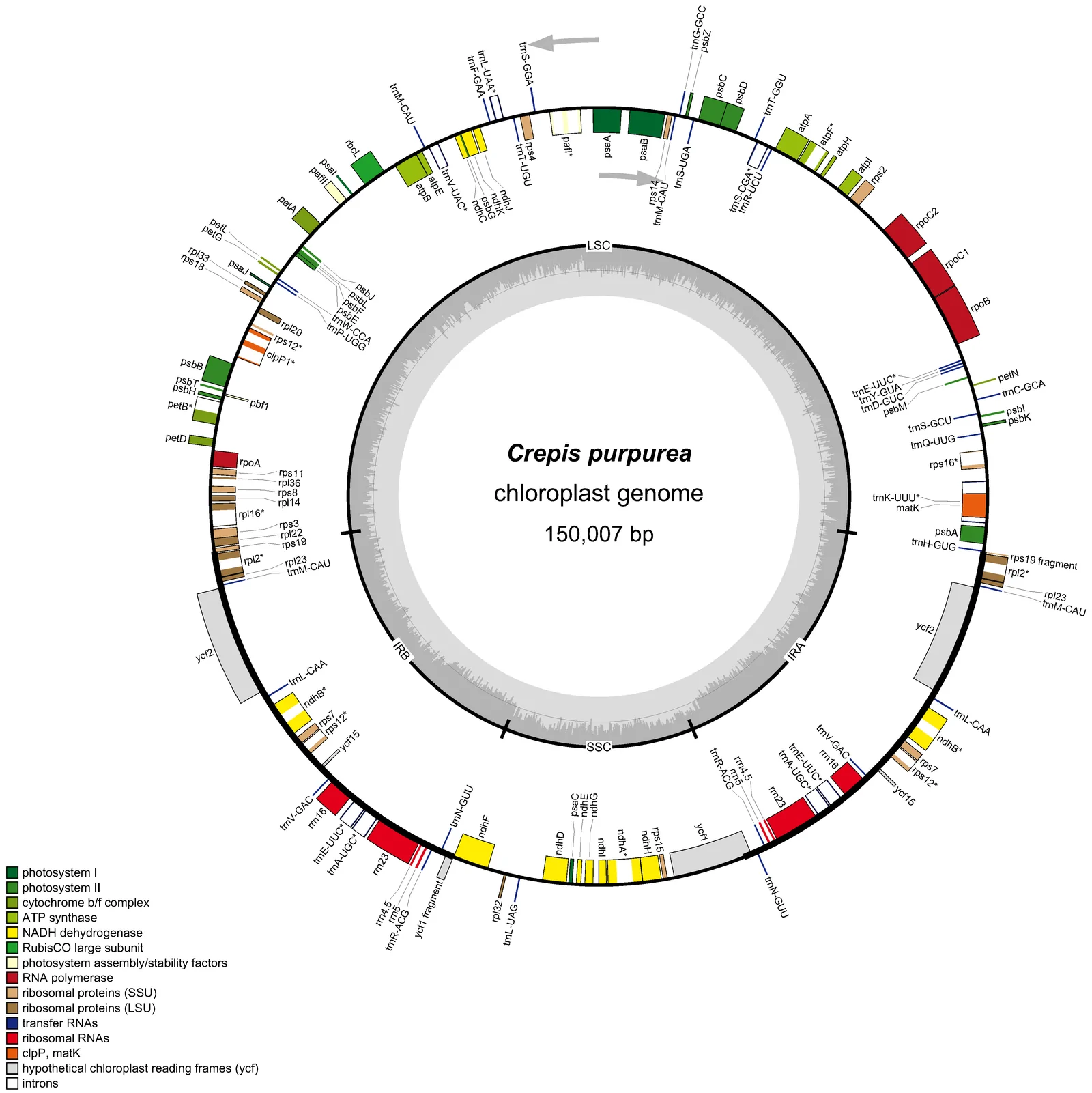
The map of the *Crepis purpurea* plastid genome. Colours indicate gene and feature categories as shown in the legend. Genes inside the circle are transcribed clockwise, whereas those outside are transcribed counterclockwise; grey arrows indicate the direction of transcription. Asterisks (*) mark intron-containing genes. The inner ring shows the local guanine–cytosine (GC) content as a dark-grey profile. LSC, large single-copy region; SSC, small single-copy region; IRA and IRB, inverted-repeat regions A and B. The disrupted *accD* locus (293 bp) between *rbcL* and *psaI* is not drawn.

As a result of *de novo* assembly of *C. purpurea* long reads, a circular sequence of 150,007 bp was obtained (Figure 3). The latter plastome showed the same structure but differed in length by 27 bp.

In the plastomes of both *Crepis* species, 114 unique genes were annotated (134 including the copies duplicated in the repeat regions), which are known to be present in plastid genomes within the Asteraceae family. These genes comprise 81 protein-coding genes (including *accD*, whose open reading frame is disrupted, possibly pseudogenised), 4 ribosomal RNA genes, and 29 transfer RNA genes. Two fragmentary features at the inverted-repeat boundaries (*rps19* and *ycf1* fragments) were not counted as separate genes. In *C. purpurea*, compared with the *C. callicephala* annotation, several tRNAs were attributed differently. The sequences of *C. callicephala* and *C. purpurea* plastomes differ by 16 substitutions and 34 indels (153 bp in total, the second inverted-repeat copy not counted; Table S2), most of which fall in non-coding regions. Eighteen of them represent 1–2 bp length differences in homopolymer runs of at least 7 bp. Within coding sequences, three substitutions and five short indels were found: a 24 bp in-frame tandem duplication in *rpoC2* and four 1 bp indels in homopolymer runs that would shift the reading frames of *rpoC1*, *rpoC2*, *psaA* and *rpl22* in *C. purpurea*. The assembled plastid genomes were deposited in GenBank at the National Center for Biotechnology Information (NCBI) under accession numbers PZ258326.1 (*C. callicephala*) and PZ336829.1 (*C. purpurea*). Both accessions are indexed under *C. purpurea* in NCBI Taxonomy (see Data Availability Statement).

### 2.2. Sequence Loss Affecting the accD Locus

**Figure 4:**
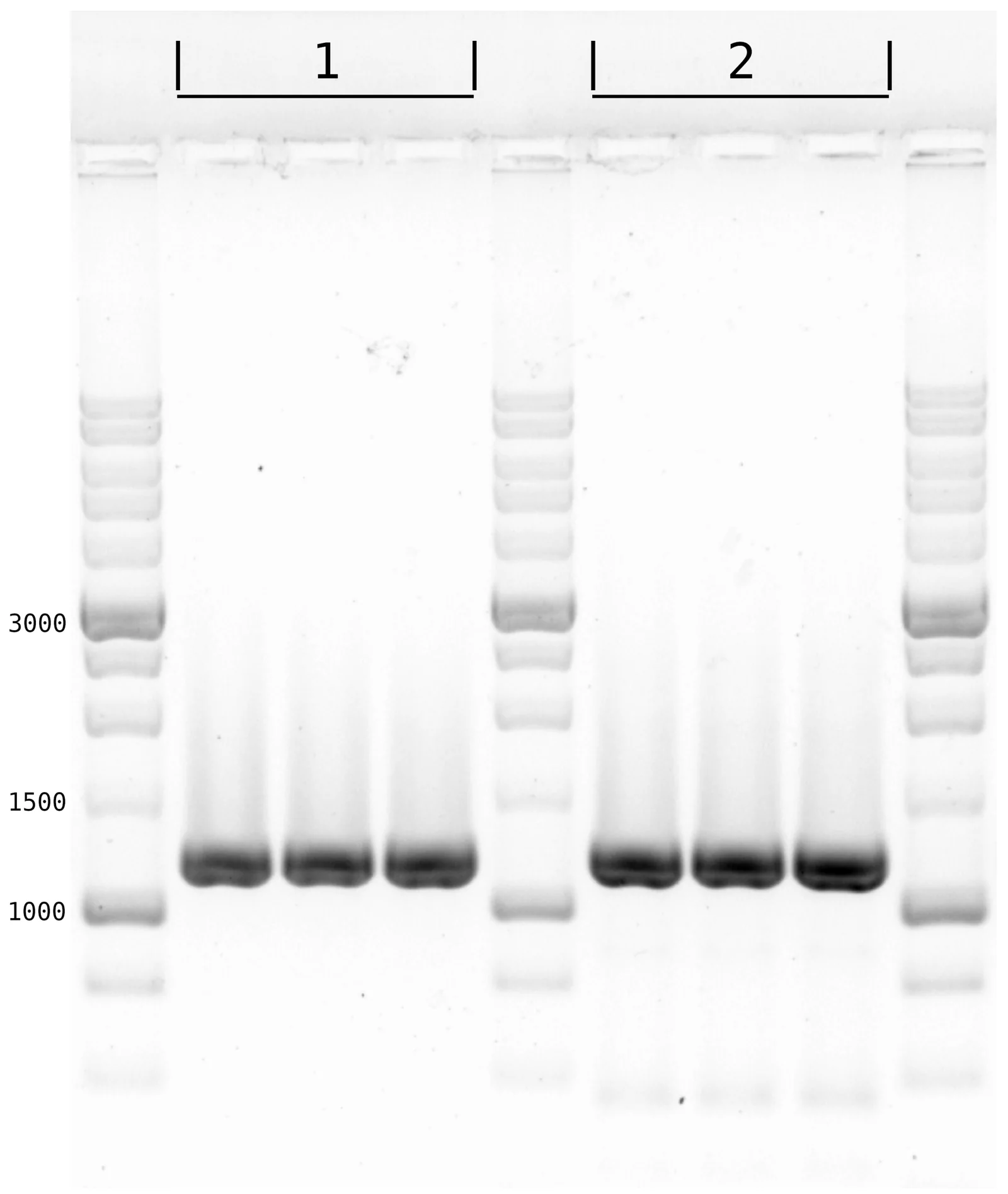
PCR amplification of the *rbcL*–*psaI* fragment with primers rbcL_01_Fwd and psaI_01_Rev, agarose gel stained with ethidium bromide. 1, *Crepis purpurea* (template DNA diluted sixfold); 2, *C. callicephala*. For each species, the same reaction mixture was loaded into three wells. Expected product sizes are given in Table 1. The outer and central lanes: DNA size marker Sky-High (Biolabmix; 250–10,000 bp); selected marker bands are labelled in bp. The lanes were cropped from a single gel; the original image is provided in Figure S1, and control reactions are shown in Figure S2.

For the *accD* gene of both species, only an annotated remnant of 293 bp was recovered, being much shorter than in the remaining Crepidinae (average ∼1478 bp; see Table S3)—a sequence containing a ∼106 bp segment homologous to an internal part of *accD* and lacking a recognisable start codon. Alignment against the *Lactuca sativa* reference plastome showed that most of the *accD* gene is not represented; a separate ∼46 bp segment homologous to the $5^{\prime }$ end of *accD* lies upstream of the annotated remnant, while the *rbcL*–*accD* spacer carries a 90 bp deletion, and a 25 bp tandem duplication is located at the junction between the $3^{\prime }$ end of *rbcL* and the spacer. The interval between the two main alignment blocks measured approximately 2.1 kbp in *L. sativa* and 250 bp in *C. callicephala*. Mapping of ONT reads to the respective plastome assemblies provided read-level support for the shortened configuration. A total of 947 unique reads in *C. callicephala* and 849 in *C. purpurea* spanned the altered *accD* locus and adjacent noncoding sequence, together with at least 100 aligned bases in each of the flanking genes, *rbcL* and *psaI*. Across the interval from the start of the upstream *rbcL* gene to the end of the downstream *psaI* gene, mean per-base coverage calculated using all reads overlapping at least part of the interval was 1629× and 1066×, respectively. PCR provided an independent experimental check of the reduced length of the *rbcL*–*psaI* fragment amplified using primers located within the two flanking genes. The expected product sizes are given in Table 1. The bands observed on the gel were approximately 1.2 kbp for both *C. callicephala* and *C. purpurea*, consistent with the corresponding predicted amplicon sizes of 1234 and 1228 bp, respectively (Figure 4). A product of the same size was also obtained from an additional *C. purpurea* plant collected from a natural population at Ak-Kaya (Figure S1). Control reactions are shown in Figure S2.

### 2.3. Inverted-Repeat Junction Site Analysis of C. callicephala, C. purpurea and Related Species

**Figure 5:**
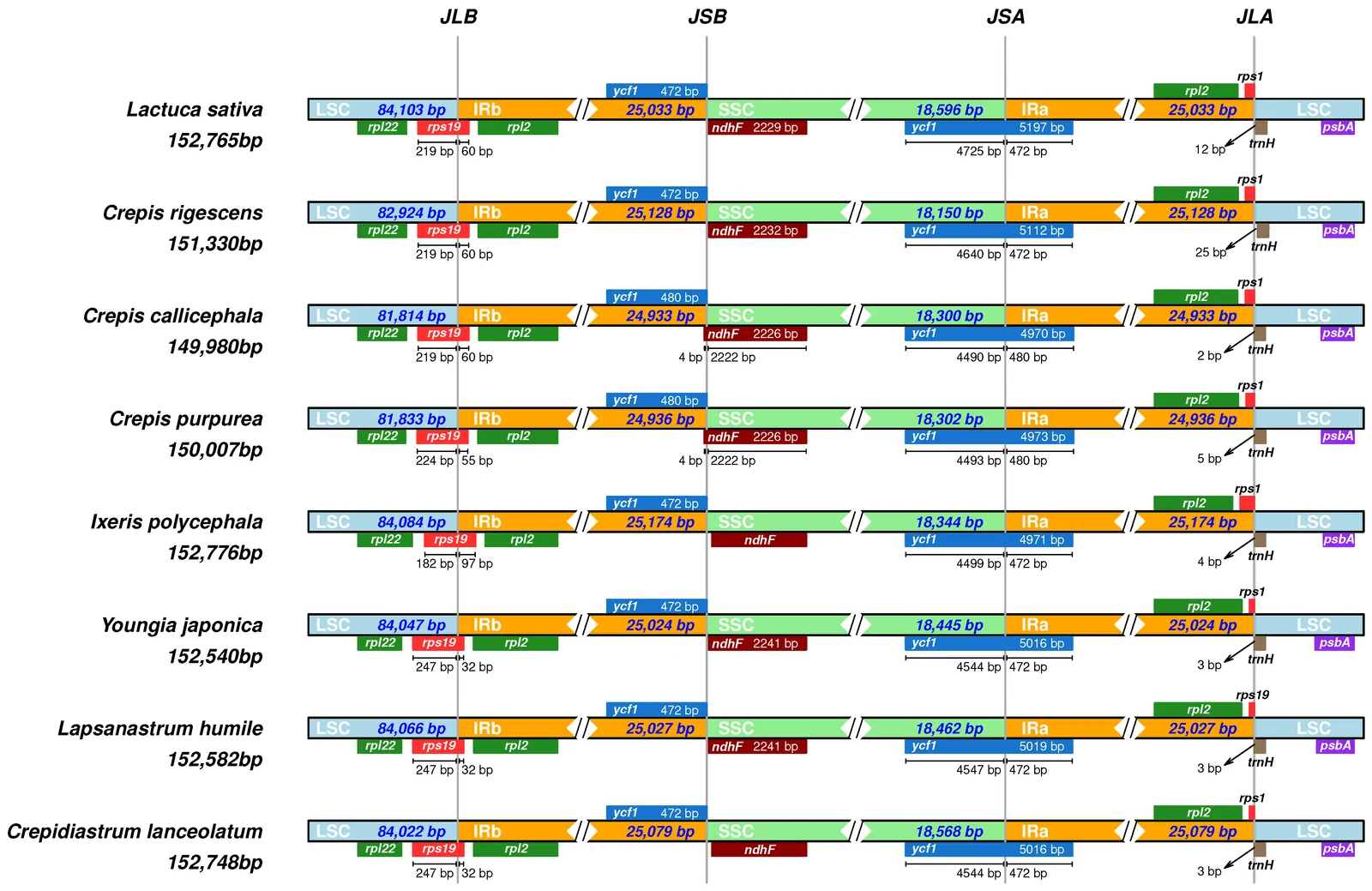
Visualisation of inverted repeats junction sites within plastomes of *Crepis* and related Crepidinae plus *Lactuca sativa*. LSC, large single-copy region; SSC, small single-copy region; IRa and IRb, inverted-repeat regions A and B. JLB (junction IRb/LSC), JSB (junction IRb/SSC), JSA (junction IRa/SSC), JLA (junction IRa/LSC).

To verify the junction sites between the large single-copy (LSC), small single-copy (SSC), and inverted-repeat (IR) regions, the surrounding loci were amplified. The two inverted-repeat copies are designated IRa and IRb. The successful amplification of all the fragments was validated by agarose gel electrophoresis, suggesting the quadripartite structure to be correctly identified (see Figure S3). To assess positions of genomic features located within junction loci of *C. callicephala* and *C. purpurea*, we compared them with those of the other *Crepis* and related Crepidinae species, as well as the outgroup (*L. sativa*). The junctions, with annotations of bordering features, are visualised in Figure 5. In both species, three genes overlap the region junctions—*ycf1* (expanding into the repeats by 480 bases), *rps19* (expanding by 55–60 bases) and *ndhF*. According to Chloë-supported GeSeq annotation, the *ndhF* gene’s $3^{\prime }$ end, starting at the SSC region, overlaps the *ycf1*-containing side of the inverted repeats by 4 bases. The LSC/IR junction position differed between *C. callicephala* and *C. purpurea*, leading to the *rps19* gene fragments bordering the junction sites being unequal: the fragments within the inverted repeats were 60 and 55 bp, respectively. The *trnH* gene was also offset from the junction site by 2 and 5 bases in the two species, in contrast to a space of 25 bp in *C. rigescens* and 12 bp in *L. sativa*. Compared with related species, *C. callicephala* and *C. purpurea* differed in that the *ndhF* gene spanned the junction site, while it remained within the SSC region in the other species under comparison. The *ycf1* gene of all the Crepidinae assessed (except for *C. callicephala* and *C. purpurea*) as well as *L. sativa* spanned the repeat regions by 472 bp, in contrast to 480 bp in the plastomes presented here.

### 2.4. Phylogenetic Analysis of C. callicephala and C. purpurea in the Crepidinae and Cichorieae Scopes

For the phylogenetic analysis, we retrieved all plastomes of Crepidinae taxa available to date from databases and included plastid genomes of four Cichorieae taxa other than Crepidinae (*Cichorium intybus* NC_043842.1, *Lactuca saligna* NC_066756.1, *Lactuca sativa* NC_007578.1, *Lactuca sativa* var. *angustana* NC_061693.1) to use as an outgroup, with filtering out duplicated sequences (Table S4). Some sequences (*Ixeris repens* NC_057108.1, *Taraxacum amplum* NC_031816.1, *T. brevicorniculatum* NC_032056.1, *T. kok-saghyz* NC_032057.1, *T. mongolicum* OL875302.1, *T. obtusifrons* NC_031815.1) differed in the orientation of genomic regions, and a sequence of another accession (*Cichorium intybus* NC_043842.1) had start position shifted by 84,305 bp while LSC region size was 84,232 bp, so that the sequence deposited in databases actually started at 73 bp of the IRb region of the properly rotated circular sequence (refer to Table S4 for annotation details). Sequences prepared for the analysis were deposited in Zenodo open repository under DOI https://doi.org/10.5281/zenodo.10989900. All the sequences alongside *C. callicephala* and *C. purpurea* plastid genomes were annotated and oriented in the way described in the Section 4, resulting in a multi-FASTA file of concatenated LSC, IRb and reverse-complemented SSC regions, with the second inverted repeat excluded. The final alignment contained 135,410 nucleotide positions, of which 13,255 were variable and 8351 were parsimony-informative, and gaps or undetermined characters accounted for 6.11% of the matrix.

**Figure 6:**
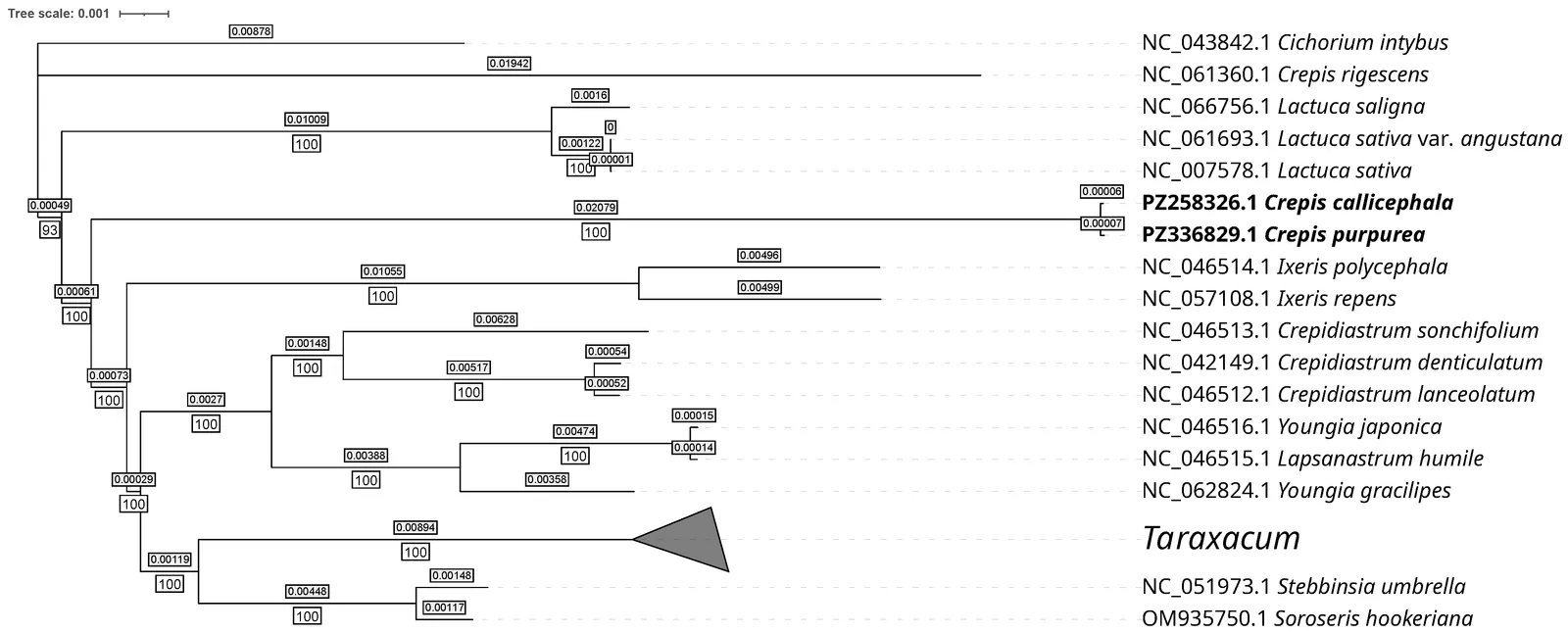
Visualisation of the Crepidinae subtribe (Cichorieae, Asteraceae) phylogenetic tree based on a multiple sequence alignment of plastome sequences, with the exclusion of the second inverted-repeat copy (IRa). The tree was reconstructed using RAxML-NG and visualised in iTOL v7, with rooting on *Cichorium intybus*. Node support values (Felsenstein bootstrap, 1000 replicates) are shown below the branches and branch lengths above them. The *Taraxacum* clade (12 accessions) was collapsed to simplify the tree visualisation, but all accessions were included in the analysis. Accessions sequenced in this study are shown in bold.

The phylogenetic tree was constructed from the multiple sequence alignment using a maximum-likelihood approach. The trees that were produced by RAxML-NG and IQ-TREE software had identical topologies (Figures S4 and S5). The tree presented in Figure 6 was computed using RAxML-NG. In both the RAxML-NG (Figure 6) and IQ-TREE (Figure S5) analyses, node support was 100 at nearly all nodes, including the node uniting *C. callicephala* and *C. purpurea*. Along the subtribal backbone, the lowest support was at the node subtending *Lactuca* and the Crepidinae (93 with the Felsenstein bootstrap in RAxML-NG and 81 with the standard bootstrap in IQ-TREE). Lower support values were confined to shallow nodes within the *Taraxacum* clade (as low as 46 and 56, respectively, Figures S4 and S5). In the RAxML-NG tree shown in Figure 6, the branch subtending the *C. callicephala*–*C. purpurea* clade was the longest (0.0208), followed by the terminal branch of *C. rigescens* (0.0194). Both were approximately twice as long as the branches subtending the *Lactuca* and *Ixeris* clades (0.0101 and 0.0105, respectively). The IQ-TREE tree showed the same pattern (0.0215 and 0.0202, respectively). The accession deposited as *C. rigescens* (NC_061360.1) fell outside the remaining Crepidinae taxa (see Section 3.5), while *C. callicephala* and *C. purpurea* branched off just before the other sampled species within the subtribe. *C. callicephala* and *C. purpurea* were separated by very short terminal branches.

**Figure 7:**
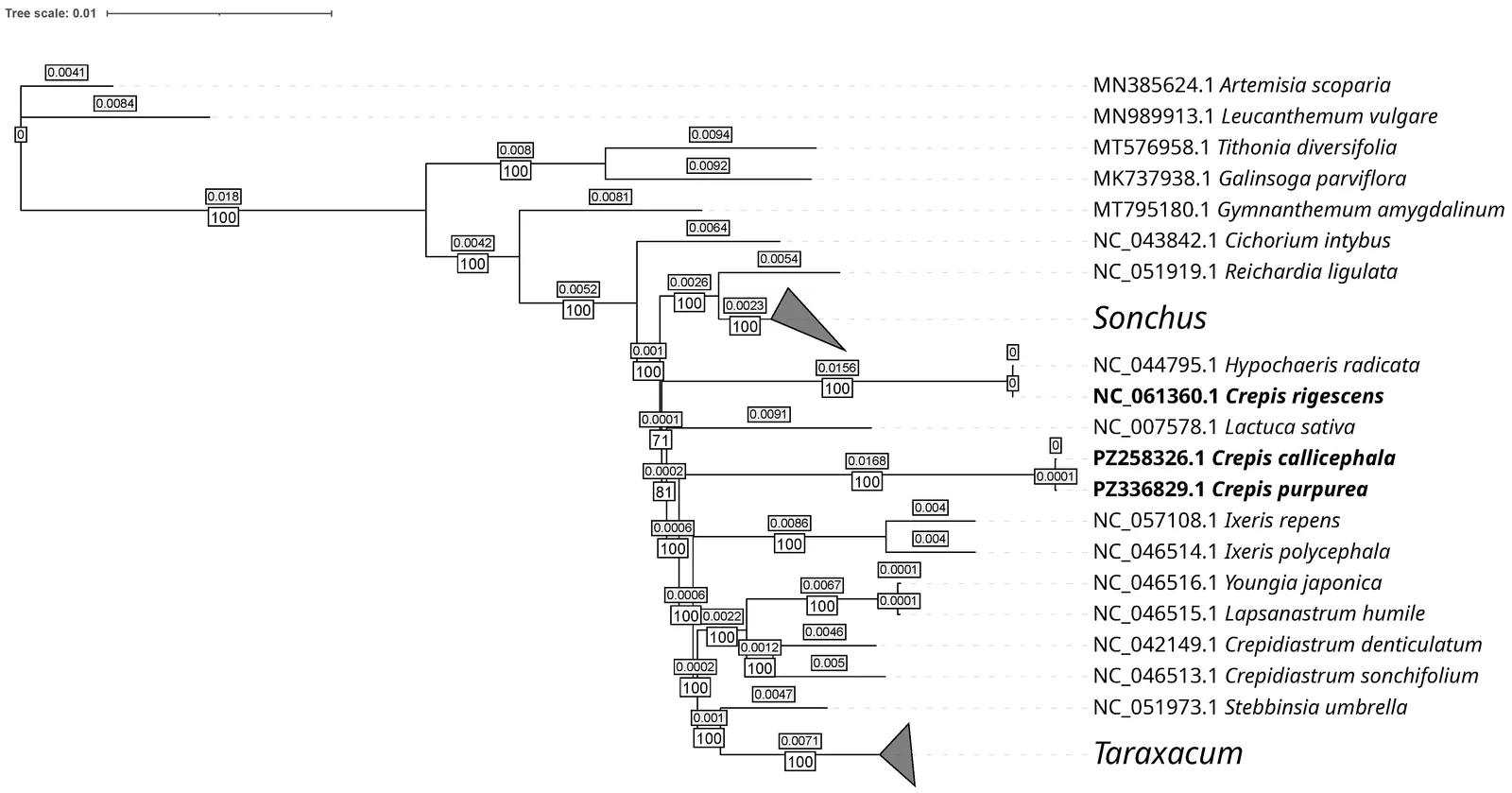
Maximum-likelihood phylogenetic tree placing *Crepis callicephala*, *C. purpurea* and the NC_061360 accession within a broader sampling of the Cichorieae tribe, based on whole-plastome sequences. The taxon set follows P. Li et al. [28]. The tree was reconstructed with RAxML and visualised in iTOL, with bootstrap values (500 replicates) shown below the branches and branch lengths above them. The *Taraxacum* (10 accessions) and *Sonchus* (13 accessions) clades were collapsed to simplify the tree visualisation, but all accessions were included in the analysis. The two accessions sequenced in this study (PZ258326.1 and PZ336829.1) and the accession deposited as *Crepis rigescens* (NC_061360.1) are highlighted in bold.

To clarify relationships among *Crepis* species and their placement within the Cichorieae tribe, we reconstructed the phylogenetic tree of the tribe utilising accessions used in the phylogenomic study of the *C. rigescens* plastome [28]. The sequences were processed as for the Crepidinae tree, whereas the alignment strategy, tree inference program and outgroup differed (Section 4.5, Figure 7). Bootstrap support was 100 for nearly all branches, including the node uniting *C. callicephala* and *C. purpurea*, and decreased only at the deep subtribal nodes, with 71 at the (NC_061360.1 + *Hypochaeris radicata*)/(*L. sativa* + Crepidinae) split and 81 at the *L. sativa*/Crepidinae split. Notably, the topology and branch lengths of the shared taxa were very similar to those of the Crepidinae tree. The longest branch within the Cichorieae subtended *C. callicephala* and *C. purpurea* (0.0168), and the second longest subtended the NC_061360.1 accession and *H. radicata*, which clustered within the Hypochaeridinae (0.0156). Aligned against *H. radicata*, the NC_061360.1 plastome was fully collinear and differed at only three nucleotide positions (Table S5). In this tree, the distance between *C. callicephala* and *C. purpurea* also remained short.

## 3. Discussion

The plastid-based phylogeny reconstruction has become one of the most commonly used methods in plant molecular phylogenetics. This is primarily due to the fact that plastids are relatively small in size, about 150 kbp on average, while being represented in a multitude of copies in the cell. Due to this, it is possible to obtain a sufficient number of sequencing reads to assemble a plastid genome even in cases of low sequencing depth. Because of the number of virtually identical molecules of the plastid genome being hundreds and thousands of times greater than the amount of DNA originating from the nuclear genome, it has become possible both to sequence a much larger number of samples in a single sequencing run and to obtain information from highly degraded material, such as museum and herbarium specimens [29].

Plastids are organelles involved in many plant cell processes, the most notable of which is photosynthesis. They possess their own DNA, characterised by some features of the prokaryotic genome, for example, type of codon coding for amino acids, cyclic topology and ribosomal RNA of prokaryotic type. The plastome of vascular plants is considered to be highly conserved and is represented by a circularised double-stranded DNA molecule comprising two prolonged regions (Large Single Copy region, LSC; Small Single Copy region, SSC) separated by inverted repeats (IR), referred to as quadripartite structure. The gene content and order among a large taxonomic group such as a family appears to be nearly the same, as in the Asteraceae family [30], which favours phylogeny reconstruction based on the full plastid DNA sequence alignment.

It is known that despite the existence of four conserved regions, the plastid genome has a natural structural variability within the cell, represented in the formation of oligomers and even linear molecules [31,32], as well as isoforms [33]. These kinds of variability complicate the assembly of the plastid genome.

### 3.1. Strategies of Plastid Genome Sequencing and Assembly

Historically, plastid genome data were obtained using Sanger sequencing, the most exact but labour-intensive approach. Now, the most common approach in plant phylogenomics has become the use of Illumina platform-derived short paired-end sequencing reads. But the presence of nuclear sequences in the set of sequences from which plastid DNA is to be assembled may complicate assembly for several reasons. Firstly, it increases computational time, and secondly, some plastome DNA sequences might share similarities with the nuclear genome. Therefore, the sequencing library is enriched with sequences of the plastid genome. Here, some approaches exist, such as hybrid capture [34], chloroplast isolation [35], long-range PCR [36,37], but they are also labour-intensive.

Another approach is to make use of the differences in copy level between nuclear and plastid DNA by sequencing huge barcoded datasets with low depth. In such libraries, only overrepresented sequences would reach an amount sufficient to assemble a complete plastid genome. This method, referred to as genome skimming, allows for the production of data for a set of samples in parallel [38,39,40].

With the rise of high-throughput sequencing technologies, a huge amount of WGS data are produced by the research community. It seems to be a great source for both nuclear and organelle genome data. This approach is computationally expensive but yields high-quality sequences. Most sequencing projects are performed using the Illumina platform. Long-read data are also being produced at a substantial level, enabling the resolution of the structural variability problem [36]. There are some efforts to assemble plastid genomes from reads produced on Pacific Biosciences platforms [41,42,43,44], but nanopore reads produced by MinION and PromethION platforms of Oxford Nanopore Technologies are still underused.

### 3.2. Putative Pseudogenisation of accD in the Plastomes Studied

In the study of K. McGrath, in particular, on the example of the hypothetical loss and preservation of exon 1 of the *rpl16* gene in different groups of Asteraceae, imperfect annotation is discussed as a source of false conclusions, while there are no objective differences between the genomic features under consideration. Conducting a comparative study of 36 species plastomes from different subfamilies and tribes within Asteraceae, Pascual-Díaz et al. concluded that plastid genomes within the family are highly conserved, possessing (with the exception of the basal branch—the Barnadesioideae subfamily) the same structural features and gene order [45], also confirming the generality within Asteraceae of two family-specific inherited inversions located in the LSC region [46]. The loss of a fragment of the *rps19* gene at the end of one of the inverted repeats is also discussed in the literature [45], however, this may be an annotation error, as in the case of the erroneously recognised loss of exon 1 and intron in the *rpl16* gene in the *Lactuca sativa* and some other Asteraceae [47]. For the core clades of a family the asymmetric use of codons within the plastid genome has been noted, being linked to threonine transfer RNA *trnT-GGU* pseudogenisation [48]. This is a rare case of radical asymmetry, spanning the entire clade, when only one threonine transfer RNA, the *trnT-UGU*, retains functionality. It was also noted that of the 13 studied genomes of species within the Cichorieae tribe, pseudogenisation was determined all the times the ARAGORN program was used, whereas the tRNAscan-SE program, that is used more often as part of GeSeq tool suite, distinguished pseudogenisation in less than a third of cases. The results obtained indicate the importance of exactly how the analysed data were obtained.

At the same time, during the study of the plastid genomes of *C. callicephala* and *C. purpurea*, we discovered a feature that does not seem to be typical of the core Asteraceae, whose plastomes are generally regarded as conserved in structure, gene content and gene order—the putative pseudogenisation of the *accD* gene. The *rbcL*–*psaI* locus of the two species is shorter than in the other Cichorieae accessions studied, and the gene boundaries determined by alignment do not match an open reading frame, suggesting changes that may have caused the loss of function of this gene.

The enzyme acetyl-CoA carboxylase (ACCase) catalyses the first stage of fatty acid biosynthesis in plants, the carboxylation of acetyl-CoA into malonyl-CoA. In plants, there are two types of such enzymes—eukaryotic homomeric ACCase, encoded in the nuclear genome, and a prokaryotic heteromeric protein consisting of four subunits, some of which are encoded in the plastid genome—the peptide of the β-carboxyltransferase (β-CT) subunit is encoded by the *accD* gene [49]. It has been shown that an increase in acetyl-CoA carboxylase activity in tobacco is associated with the β subunit of acetyltransferase, and directly correlates with the expression of the *accD* gene [50]. Moreover, the leaves of the transformed tobacco plants showed a significant increase in both fat content and leaf length. At the same time, the fat content in the seeds changed slightly, but each plant produced twice as many seeds, which can be reduced to a two-fold increase in per-plant fat production. A similar effect of the dependence of fat content on the expression level of the plastid gene *accD* and nuclear *accC* was shown for the oil palm (*Elaeis guineensis* Jacq.) [51]. Thus, the *accD* gene affects the quality and quantity of seeds produced by the plant.

Functional experiments on the tobacco model have shown that acetyl-CoA carboxylase is of critical importance for normal plastid function. Thus, knockout of the *accD* gene in the plastid genome led to the development of different forms of defective leaves with mosaicism and inclusion of etiolated areas due to heteroplasmy, as well as abnormalities in the plastid structure [52]. Decline in the expression of the tobacco *accD* gene due to the introduction of a knockdown mutation in the start codon site was phenotypically manifested in a decrease in growth rate, a violation in plastid division, a decrease in seed production and other effects [53].

RNA editing is a frequent processing event in plastid DNA and might affect coding sequences. One of the striking examples is the replacement of the second nucleotide in the UCG triplet (serine) with UUG (leucine) in the pea *accD* gene, which helps restore acetyl-CoA carboxylase activity for plants that have a non-functional variant with the UCG codon [54]. Several editing sites have been found in the transcript of *Arabidopsis accD* gene, in particular, C794 in the exon and C1568 in the $3^{\prime }$ non-coding sequence [55]. The mutation leading to editing breakage in *accD*-C794 site affects the activity of acetyl-CoA carboxylase, which became apparent in the form of chlorosis and abnormalities in the ultrastructure of plastid membranes that appear under thermal stress conditions, and is biochemically manifested in a reduction in unsaturated oleic fatty acids (C18:1) and linoleic acids (C18:2) content [56].

It has been established that genes encoding peptides of ACCase subunits in the plastid genome are involved in the activation of lipid accumulation by exposure to light [57].

With the rise of sequencing technologies, it has become clear that the transfer of DNA sequences from plastid to nuclear genome is a fairly common phenomenon in the plant world, involving primarily intergenic regions [58]. There are several cases of migration of genes encoding acetyl-CoA carboxylase between genomic compartments in higher plants. In particular, it is known that in *Gnetum*, the *accD* gene was transferred to the nucleus during two independent events, forming two independent nr-*accD* genes [59]. The transition of *accD* to the nucleus was also noted for Campanulaceae, in which nr-*accD* turned out to be significantly shorter than the original plastid gene, including only the carboxylase domain with a small fragment of surrounding sequence. Moreover, both genes are expressed and their products are found in plastids [60]. Not all the taxa in which *accD* was transferred to the nucleus retained the plastid gene intact. In *Primula sinensis* (Primulaceae), a deletion of 400 bp in size was reported in the gene, affecting a conserved functional region, whereas no similar pseudogenisation was found in other representatives of Primulaceae [61]. The most notorious example of the loss of the *accD* gene is the Poales clade, in a number of whose members the pseudogenisation or complete loss of this gene has occurred [62]. In particular, it was noted that cereals lack the functional gene of the β-subunit of acetyl-CoA carboxylase but possess at the same time the gene for the homomeric enzyme [63], and the presence of its products in plastids was experimentally established [64].

The lack of heteromeric acetyl-CoA carboxylase is associated with the selective clade-specific sensitivity of cereals to a number of herbicides [63,65]; however, there are certain mutations in homomeric acetyl-CoA carboxylase gene sequence known to be linked to the resistance against carboxylase-targeted herbicides [66,67,68].

The phenomenon of nuclear cytoplasmic conflict in peas is supposed to be associated with interaction between the subunits of heteromeric acetyl-CoA carboxylase [69].

The tobacco experiments demonstrate that disruption of plastid *accD* impairs normal development in that species [52,53]. The AccD protein is one of four subunits of heteromeric ACCase, so disruption of its plastid-encoded gene raises a question about the function of the enzyme complex as a whole. The examples discussed above show, however, that the absence of an intact *accD* gene in the plastid genome need not entail loss of ACCase activity in plastids. Possible mechanisms include a functional nuclear *accD* copy whose product is imported into plastids, or a plastid-targeted, nuclear-encoded homomeric ACCase [59,60,63,64]. The functional consequences of the altered locus in the two *Crepis* species studied here remain to be determined.

A comparable loss of *accD* and adjacent noncoding sequence has not been reported in other members of Cichorioideae. A different alteration of *accD* was recently described in *Adenocaulon himalaicum* (Asteraceae, subfamily Mutisioideae): the authors annotated *accD* as a pseudogene and attributed the predicted premature termination to a repeat insertion near the $3^{\prime }$ end of the gene [70]. The plastome of *Taraxacum hallaisanense* (Cichorioideae) has also been reported to contain an *accD* pseudogene, although the underlying sequence change was not described [71]. Several database records likewise carry *accD* pseudogene annotations: a partial plastid sequence of *Youngia japonica* (KM192088.1) and the plastomes of *Ixeris polycephala* (NC_046514.1), *Lapsanastrum humile* (NC_046515.1) and *Y. japonica* (NC_046516.1). By contrast, an *accD* coding sequence is annotated in *Y. gracilipes* (NC_062824.1) and *I. repens* (NC_057108.1). Another accession listed among *Crepis* plastomes, NC_061360.1, also has an annotated *accD* coding sequence, but cannot serve as a reliable *Crepis* comparison: although deposited as *C. rigescens*, its plastome is nearly identical to that of *Hypochaeris radicata* (Section 3.5).

By careful analysis we discovered that the *accD* locus is markedly altered in the species under investigation: the gene body is largely missing together with adjacent noncoding sequence, and no intact plastid *accD* open reading frame can be reconstructed, while smaller deletions and insertions are also present nearby. Some complex recombination might have occurred in this locus of the plastid genome. We suppose two scenarios are possible: (1) recombination with transfer of part of the *accD* gene sequence to the nuclear genome, or (2) compensation for the putative loss of plastid-encoded AccD function by a nucleus-encoded homomeric enzyme. The available plastome data do not distinguish between these scenarios.

The expression pattern of *accD* in *Crepis* species, in particular in the *Lagoseris* lineage, the search for its products, as well as the machinery of ACCase action and delivery, need further study.

### 3.3. Approaches for Phylogeny Reconstruction Using Plastid Genome Data

Several research directions in plastid-based phylogeny reconstruction have historically developed over a period of more than thirty years. One of the oldest and most obvious techniques relied on the study of short sequences of polymorphic loci in plastid DNA, amplified by PCR and most often sequenced by Sanger. Early large-scale molecular phylogenetic studies of angiosperms combined plastid loci *rbcL* and *atpB* with nuclear 18S rDNA [72]. A subsequent study expanded this three-gene matrix by adding plastid *matK* and nuclear 26S rDNA [73]. Molecular phylogenetic studies of this kind provided evidence for successive Angiosperm Phylogeny Group (APG) classifications of the orders and families of flowering plants [74]. Plastid DNA and nuclear ribosomal DNA occur at high copy number and can therefore be readily recovered by genome skimming [40]. Plastid phylogenies usually trace a single, predominantly uniparentally inherited lineage, whereas nuclear ribosomal DNA occurs in tandem arrays and may contain divergent paralogous copies [75,76].

However, single protein-coding loci may provide an insufficient phylogenetic signal for comparisons among closely related taxa. This limitation has been demonstrated for *matK* in closely related pines [77], despite its higher variability compared with *rbcL* and *atpB* [78]. Since coding sequences are generally less variable than plastid introns and intergenic spacers, a set of noncoding loci widely used in plant phylogenetic studies has gradually become established. Shaw and co-authors compared the informativeness of 21 such loci from the LSC across major lineages of seed plants, while also assessing the universality of available primers [79].

At the same time, with the development of high-performance sequencing techniques, a large number of complete plastid sequences have appeared, which has opened up opportunities for two types of comparative analysis: one is based on a set of protein-coding sequences or proteins [28,45,80] and the second on complete sequences of the plastid genome; moreover, the latter approach is used more often.

### 3.4. Overcoming the Non-Uniformity of Database-Derived Plastome Data in Phylogeny Reconstruction

In the plastid genome of *Lactuca sativa* (GenBank id: NC_007578), protein-coding sequences occupy 47.8% and non-coding sequences 44.5%; in *C. callicephala*, the corresponding values are 51.7% and 40.4%. A high synteny is known to be observed when comparing plastomes within the same family, and non-coding sites give a strong phylogenetic signal; therefore, the use of complete sequences can be informative when analysing both closely related and distant species.

For this purpose, the plastome sequence is commonly compared without one of the inverted repeats (the two copies are nearly identical and reverse-complementary to each other) [45]. Moreover, if a standardised isoform of plastid DNA is used—that is, the sequence of the LSC-IRb-SSC-IRa regions—and the second inverted repeat is then discarded, the *ycf1* gene will appear to overlap the boundary between the repeat and the SSC and be divided into two fragments—one will remain at the end of the repeat, and the other at the end of the SSC. If, in addition to excluding IRa, the SSC region is inverted, the gene sequence remains intact. This approach is sometimes used in phylogenetic comparison of plastid genomes, and we also followed it by first bringing the sequence of the plastome to the appropriate form. Thus, both genes along which the inverted repeat boundary lies—*rps19* and *ycf1*—are fully represented in the alignment (Section 4.5).

### 3.5. Crepidinae Subtribe Phylogeny Reconstruction and the Position of the Lagoseris Clade

In the Crepidinae tree (Figure 6), the accession deposited as *C. rigescens* (NC_061360.1) formed a markedly long branch and diverged from the remaining Crepidinae even before *Lactuca* (Lactucinae). To clarify the position of *Crepis* species within the Cichorieae tribe, we performed a phylogenetic reconstruction using the same accessions as in the publication of P. Li and co-authors [28] where the first plastid genome-based phylogeny reconstruction of *C. rigescens* was presented. The authors performed multiple sequence alignment of fifteen proteins encoded in the plastid genome, but did not specify which ones were used in the analysis. We similarly aligned the complete sequences of the plastid genome and observed the same branch topology (Figure 7), in which the *Crepis* accession previously reported by P. Li et al. (NC_061360.1) clustered very close to *Hypochaeris radicata* (NC_044795.1) from the sister subtribe Hypochaeridinae, with lower support at the deep subtribal nodes (Section 2.4). Outgroups selected stepwise at the levels of closely related genera, subtribe, tribe and subfamily support the correct placement of NC_061360.1 (deposited by P. Li et al. as *Crepis rigescens* L.), minimising the impact of long-branch attraction; both accessions fell on essentially the same branch. Direct comparison confirmed that the two plastomes are nearly identical, fully collinear and differing at only three nucleotide positions (in *rpoB*, an intergenic region, and *rps12*). Such minimal divergence is far below the variation expected between two independently collected individuals—and is incompatible with two different genera—indicating that both records originate from the same plant material, the few differences most likely reflecting assembly or sequencing artefacts. NC_061360.1 therefore represents another instance of the *H. radicata* plastid genome rather than an independent *C. rigescens* plastome. Consequently, the *C. callicephala* plastid genome (PZ258326.1) may be considered a useful reference point for further plastid-genome studies within the genus *Crepis*, particularly for comparisons within the *Lagoseris* lineage. Several questions arise in light of such a topology. The first one concerns the position of the branches of the genus *Crepis* within the tribe Cichorieae and the status of *Crepis* as a typical genus for the Crepidinae subtribe. The second question might be phrased more narrowly—what are the phylogenetic relationships between *Crepis* and *Lactuca*. The third question concerns the generic identity of the NC_061360 sample, which was sequenced under the name *C. rigescens*. The fourth question is whether there is enough data for the position of the genus *Crepis* in the tribe Cichorieae to reconstruct with high confidence. And the fifth is the question of the taxonomic status of the *Lagoseris* lineage.

It should be said that the separate position of the *Lagoseris* branch within the genus *Crepis* has been discussed in a number of papers. In the classical treatment of the taxonomy of this species-rich genus (over 200 taxa of the species rank), E. Babcock places the *Lagoseris* lineage inside the genus *Crepis*. The conclusion about the existence of several main groups within the genus—among them the *Crepis sensu stricto* and *Lagoseris* lineages—has been confirmed using molecular methods [13]. This was also reflected in the revision of sections within the *Crepis* genus performed by N. Enke, in which cytogenetic and molecular data were summarised, and the *Lagoseris* clade was considered to be one of the earliest branches diverged [81]. More recent works also confirm this division into two clades [82]. Cherepanov, like Bobrov and Tzvelev in the Flora of the USSR, considers the taxa included in the *Lagoseris* clade within the scope of the separate genus *Lagoseris* M. Bieb. At the same time, H. Inceer, discussing similarities in the achene anatomy and stomatal features within the genus *Crepis sensu lato*, expresses the opinion that there are still no convincing arguments for excluding the group of species belonging to the clade *Lagoseris* from the genus *Crepis* [83].

These unresolved relationships raise once again the question of the position of *Crepis* relative to other groups within the Cichorieae tribe and underscore the need to accumulate more genomic data to resolve the phylogeny of this group. To our knowledge, at the time of this study only two *Crepis* plastid genomes other than those reported here had been described in the literature—that deposited as *C. rigescens* (NC_061360.1, 151,330 bp) from the flora of China [28], and that of the North American *C. monticola* Coville (152,715 bp) [47]. The sequence of the latter has not been deposited in databases and is known only from K. McGrath’s master’s thesis. Additional complete *Crepis* plastomes have since been deposited in public databases.

### 3.6. Contribution of Plastid Phylogenomics to the Relationships Between C. callicephala and C. purpurea

The content and order of genes in the plastid genomes of *C. callicephala* and *C. purpurea* appear to be similar, and they also share the same branch in the phylogenetic tree reconstructed. The 16 substitutions and 34 indels distinguishing the two plastomes studied (Table S2) may be consistent with the geographical, altitudinal and phenological separation of the two taxa. The low plastid divergence (approximately 1.3 × 10^−4^ substitutions per site) and the very short terminal branches of the two accessions (Figure 6) suggest a recent split of the sampled plastid lineages, but an absolute divergence time cannot be reliably estimated from a single accession per taxon using secondary calibrations. At the same time, their plastome sizes differ by 27 nucleotides. Such differences may have both biological and technical causes, since the plastid genome of *C. purpurea* was assembled from long reads, which may introduce platform-specific bias into the final assembly, such as incorrect homopolymer lengths and a non-random ratio of substitution errors of different classes [84]. The 18 indels in homopolymer runs (Section 2.1) are the most likely candidates for such artefacts, whereas the substitutions and the remaining 16 indels are less affected by this bias. In addition, differences at the junctions of the LSC region and inverted repeats indicate small shifts in the LSC/IR boundaries. However, the plastome represents a single, predominantly maternally inherited lineage, and only one accession per taxon was analysed. The observed differences therefore characterise the sampled plastid genomes and cannot by themselves delimit the species. Moreover, nuclear data would be required for this purpose. To assess the taxonomic significance of these features, plastome variation should be examined across additional populations of *C. callicephala* and *C. purpurea*, including populations from which plants were previously collected and identified as *L. robusta* under historical treatments.

## 4. Materials and Methods

### 4.1. Plant Material

The study included a single seed-derived accession of each species, *Crepis callicephala* Juz. and *C. purpurea* (Willd.) M. Bieb., with each accession originating from a single seedling. The germplasm originated from seeds collected in 2016 from natural populations in the Nikitskaya Yayla (*C. callicephala*) and the Belbek River valley (*C. purpurea*). At the Nikita Botanical Gardens (Yalta), the seeds were introduced into aseptic culture following the previously described procedure [85]. Adventitious shoots were obtained directly from the resulting *in vitro* seedlings, without an intervening callus phase, using a previously reported regeneration approach [86].

During the first year of active *in vitro* cultivation, each lineage underwent 5–6 subculture passages. The cultures were subsequently deposited in the *in vitro* genebank under slow-growth conditions, an approach previously described for *C. purpurea* [27]. In the genebank, the material was maintained for 3–4 years and subcultured once per year. Cultures were periodically returned to active cultivation from the genebank as required. The material analysed for each species represented a single clonal lineage derived from the corresponding original seedling.

High-molecular-weight DNA was extracted from young leaves of *in vitro* plantlets following the Doyle & Doyle cetyltrimethylammonium bromide (CTAB)-based protocol, with the addition of 2% (*w*/*v*) polyvinylpyrrolidone K 40 and 2% (*v*/*v*) 2-mercaptoethanol to the extraction buffer, and performing RNase A treatment and an additional washing step with 70% ethanol solution after precipitation. For flanking and PCR amplification of plastid genome fragments of interest during posterior analysis, DNA of *C. purpurea* was obtained from roots fixed in Clarke’s solution (ethanol:acetic acid glacial 3:1) for 2 h and subsequently washed in four volumes of 80% ethanol solution. DNA from roots was extracted following a sodium dodecyl sulfate (SDS)-based protocol with the addition of 0.45% (*w*/*v*) sodium metabisulfite.

Extracted DNA quantity and quality were assessed via NanoPhotometer (Implen, Munich, Germany), and fragment size via 4200 TapeStation system (Agilent Technologies, Santa Clara, CA, USA).

### 4.2. DNA Sequencing

*C. callicephala* total genomic DNA was sequenced on the Illumina and ONT platforms, while that of *C. purpurea* was sequenced on the ONT platform only.

Paired-end short-read sequencing for 300 cycles was carried out in the NextSeq 550 instrument (Illumina, San Diego, CA, USA) at the “Genomics” Core Facilities of the Institute of Cytology and Genetics, Siberian Branch of the Russian Academy of Sciences (ICG SB RAS).

Long-read nanopore sequencing was conducted at the facilities of the Kurchatov Genomic Centre—Nikita Botanical Gardens (KGC—NBG NSC of RAS). Genomic libraries were prepared from high-molecular-weight DNA using SQK-LSK109 chemistry, following the manufacturer’s protocol, except that ligating was extended from 45 min to 2 h. The sequencing was performed on the MinION instrument (Oxford Nanopore Technologies, Oxford, UK) using FLO-MIN106d flow cells.

### 4.3. Data Processing and Plastome Assembly

To call bases from the raw signals outputted by the ONT MinION instrument, the proprietary ONT Guppy v5.0.11 software was used with the sup accuracy class model dna_r9.4.1_450bps_sup, with both end and middle adapter trimming activated. To evaluate the quality of adapter trimming, the Porechop v0.2.4 tool was used [87]. Length filtering was performed using bioawk v1.0 package (https://github.com/lh3/bioawk (accessed on 11 May 2023)).

For short reads, adapter cutting and ends trimming by quality were performed by Atria v3.2.1 software [88].

To assemble the plastomes, several approaches were implemented. For short-read data GetOrganelle v1.7.6.1 assembly toolkit was used [89]. The assembly sequence obtained was further polished by Polypolish v0.5.0 [90] and POLCA (from MaSuRCA v4.1.2) tools [91].

For long-read data two approaches were assessed: reference-guided assembly and *de novo* long-read assembly. In addition, the short-read assembly, for *C. callicephala* the reference-guided assembly with long-read data was performed. The plastome-specific reads were extracted from the total sequenced DNA pool of *C. callicephala* by mapping to the *Lactuca sativa* L. reference plastome (GenBank accession ID NC_007578) using winnowmap2 v2.0.3 [92,93]. Two subsequent reference-guided assemblies were performed by miniasm-based [94] mini_assemble tool from the pomoxis v0.3.10 package (https://github.com/nanoporetech/pomoxis (accessed on 13 December 2022)), using *L. sativa* as the initial reference and the output of the first stage as the reference for the second round. To validate quadripartite structure, the sequence outputted by mini_assemble tool was annotated in the GeSeq v2.03 server from Chlorobox toolset [95], which is necessary for the junctions and genome regions orientation checking by cpdna_standardisation.py script [96]. The properly oriented sequence was then polished using the Medaka v1.6.0 tool (https://github.com/nanoporetech/medaka (accessed on 24 November 2022)) with unfiltered long reads and a model corresponding to that used for base calling. The two *C. callicephala* assemblies (short-read and long-read) were compared with QUAST v5.2.0 [97], with the short-read assembly used as the reference.

The *C. purpurea* plastid genome was assembled from self-corrected long reads only, following the *de novo* approach. In order to decrease computational load at the correction stage, the subset of plastome-derived reads was selected from the total DNA pool by aligning against the short-read-based *C. callicephala* plastome sequence using minimap2 v2.24 with -Y flag, which disables hard clipping of sequences characterised by low map quality [98,99]. These selected reads were then corrected with Canu v2.2 [100] and further assembled into a graph structure with Flye v2.9.1 [101]. The sequence path within the graph was visually curated and extracted using Bandage v0.9.0 [102]. Finally, the properly oriented plastome sequence was polished with the uncorrected long reads using the Medaka tool. The newly assembled plastid genome sequences were deposited in NCBI GenBank under accession numbers PZ258326.1 and PZ336829.1.

### 4.4. Assembled Plastome Analysis and Annotating

To bring all sequences under processing in line with a circular genome model, plastid genomic regions were annotated and adjusted by shifting and orienting. For this purpose, two approaches were used: (a) annotating regions in GeSeq and processing the resulting GenBank-formatted file with the cpdna_standardisation.py script, and (b) running the validate.py program of the NOVOWrap v1.20 tool [103].

Properly oriented plastomes were automatically annotated in GeSeq v2.03 using ARAGORN v1.2.38, Chloë v0.1.0, HMMER v3.3.1 and BLAT v36 searches, with all 31 manually checked Cichorieae plastomes available on the server chosen as references (for detailed list of accessions refer to Table S6). The GenBank-formatted files produced were manually checked in the Unipro UGENE v45.0 genome browser [104]. Missing and wrongly filled metadata fields were fixed using Biopython v1.86 [105], and annotations were visualised in OGDRAW v1.3.1 [106]. Subsequent analyses were performed using Biopython v1.86 and pandas v2.3.3 packages [107,108].

To detect short variants among assembled plastomes, pairwise alignments were performed using minimap2 [98], and variants were called with paftools from the same package [98]. Annotations were transferred using vcf-annotator v0.7 (https://github.com/rpetit3/vcf-annotator (accessed on 20 March 2024)).

To inspect the structural rearrangements in *rbcL*–*psaI* loci of *C. callicephala* and *C. purpurea* plastomes, the sequences spanning from 200 bp before the *rbcL* gene position to 200 bp after *psaI* gene were extracted and aligned against the same fragment of *L. sativa* reference plastome using GSAlign v1.0.22 [109]. Sequence homology was additionally assessed with BLASTN (BLAST+ v2.16.0, -task blastn, E-value ≤ 0.001) [110,111] against the *accD* sequences of *L. sativa* and *T. brevicorniculatum*, both among the reference plastomes used for GeSeq annotation (Table S6). To evaluate read-level support for the shortened configuration, ONT reads from each species were mapped to the respective plastome assembly using minimap2 with the map-ont preset [98]. For coverage calculations, primary and supplementary alignments sharing a read identifier were combined so that the same read was counted no more than once at any position. Mean per-base coverage was calculated across the interval from the start of *rbcL* to the end of *psaI* using alignments with a mapping quality (MAPQ) of at least 20. A read was classified as spanning the altered locus when its primary alignment had MAPQ ≥20, extended at least 100 reference positions into both *rbcL* and *psaI*, with at least 100 bases actually aligned within each gene, and contained no insertion, deletion or skipped region ≥50 bp across the intervening sequence and the adjacent 100 bp portions of the two genes.

### 4.5. Phylogenetic Analysis

To compare *C. callicephala* and *C. purpurea* plastomes with data on other accessions of the Crepidinae subtribe (Cichorieae, Asteraceae), GenBank-formatted files containing sequences and irregularly available annotations were fetched from NCBI databases via the Entrez application programming interface using Bio.Entrez package of Biopython [112,113]. In cases where the same sequences are present in separate databases, those from GenBank or RefSeq (if available) were kept [114,115]. When there was a duplicating deposition of the same sequence to the same database under different names, the revised and most recent entry was retained. The 28 accessions that were kept alongside four outgroup accessions are listed in Table S4. The completeness of genomic feature annotations and inverted regions demarcation was analysed by parsing with Biopython v1.86 and summarising with pandas v2.3.3. Improper plastid genomic region annotations and shifted start positions were fixed using the validate.py program from NOVOWrap.

For phylogenetic analysis of whole plastome sequences, the LSC region, IRb, and the reverse-complemented SSC region were concatenated, while the other inverted-repeat copy (IRa) was excluded. Only one IR copy was retained because including both near-identical copies could complicate sequence alignment, overrepresent the phylogenetic signal from the IR, and thereby bias the analysis. Processed sequences were deposited in the Zenodo open repository under DOI https://doi.org/10.5281/zenodo.10989900. Multiple plastome sequences were aligned with MAFFT v7.520 using the automatic algorithm selection (--auto) [116]. The alignment included 29 plastome sequences. Missing data were not imputed, and no alignment columns were removed or filtered before phylogenetic analysis. For the RAxML-NG v1.1.0 analysis [117], the TVM+I+G4 model was selected by ModelTest-NG (as bundled with raxmlGUI 2.0.10) [118], and for IQ-TREE v2.2.5 [119], the 12.12+I+R4 model was selected by ModelFinder [120]. The RAxML-NG tree search was started from 20 trees (10 random and 10 parsimony-based). Branch support was assessed with the Felsenstein bootstrap (1000 replicates) in RAxML-NG and with the standard non-parametric bootstrap (1000 replicates) in IQ-TREE. The maximum-likelihood topologies were inferred as unrooted and further were rooted for presentation on *Cichorium intybus* (NC_043842.1) as the outgroup, and the trees were visualised in iTOL v7 [121].

To compare the data obtained with those from the *Crepis rigescens* study within the broader scope of the Cichorieae tribe [28], the accessions used in that study were downloaded and processed in the same manner as the Crepidinae accessions (Table S7). The concatenated sequences were aligned with MAFFT using the FFT-NS-2 strategy [116], yielding an alignment of 42 sequences and 140,169 columns (9.38% gaps). The tree was constructed using RAxML v8.2.12 in raxmlGUI 2.0 [122,123] with the GTR+I+G4 model, which was preferred by ModelTest-NG under the Akaike information criterion (AIC) and its small-sample correction (AICc) [118], and 500 bootstrap replicates. Four Asteroideae taxa (*Artemisia scoparia*, *Galinsoga parviflora*, *Leucanthemum vulgare* and *Tithonia diversifolia*) were specified as the outgroup. The processed sequences are available under DOI https://doi.org/10.5281/zenodo.10989900.

### 4.6. Experimental Validation of Analysis Findings

To validate the quadripartite structure of the assembled plastomes, regions overlapping the junction sites were amplified. To check experimentally the reduced length of the *rbcL*–*psaI* region inferred *in silico*, this region, including the altered *accD* locus and the adjacent intergenic spacers, was amplified in both species using primers located within *rbcL* and *psaI* (Table 2). The same primers were also applied to DNA from an additional *C. purpurea* plant collected from a natural population at Ak-Kaya (leaf tissue dried in silica gel) and to DNA from *C. sibirica* and *Leontodon autumnalis* plants collected from natural populations in the Novosibirsk area. Due to primer annealing sites the fragment to be amplified is shorter than that used for alignment with GSAlign.

Primer sequences were developed using Primer-BLAST [124] and validated in NCBI and the Primer3 functionality of Unipro UGENE [125,126]. Primer sequences are presented in Table 2. To develop primers for junction sites, the inverted repeat overlapping fragment sequences were first flanked *in silico* using BLAST+ v2.16.0, called from a Biopython script using the subprocess module in Python 3.11, and then primers were mined and checked for cross-amplifiability [110,111].

Primer oligonucleotides were commercially produced by Biosset (Novosibirsk, Russia). To amplify DNA fragments, modified Pfu-SE polymerase and ready-to-go master mixes Biomaster HS-Taq and Biomaster LR HS (Biolabmix, Novosibirsk, Russia) were used. Phoresis was performed in 2% agarose gel (Dia-M, Moscow, Russia) for short amplicons and in 1.5% gel for longer fragments with fresh 1x Tris-Acetate-EDTA buffer. Amplicons were visualised in the Gel Doc XR+ system (Bio-Rad Laboratories, Hercules, CA, USA) and excised from the gel following marks made under the transilluminator. Gel bricks were placed inside the upper part of the pipette tips right above the filter, frozen at −18 ^∘^C, and the buffer containing DNA amplicons was isolated by centrifuging at 16,000× *g* for 5 min. DNA was concentrated by adding 10 μL of 10 M ammonium acetate to 50 μL of buffer and precipitating with 120 μL of ice-cold 95% ethanol at 16,000× *g* for 10 min, followed by washing with 70% ethanol and centrifuging for 5 min.

## 5. Conclusions

The sequenced and assembled plastid genomes of *Crepis callicephala* and *C. purpurea* are among the first plastomes published within the *Lagoseris* lineage. Comparison of the complete plastomes brought more clarity to the interrelationships among *Crepis* and related genera. In both plastomes, the *rbcL*–*psaI* interval is markedly shortened, with sequence loss affecting *accD* and adjacent noncoding DNA. Only limited *accD*-homologous sequence remains, and no intact plastid *accD* open reading frame is present. This is consistent with the pseudogenisation of plastid *accD*. The consequences for plastid ACCase activity remain unresolved. Whether this pattern occurs in other taxa within the *Lagoseris* clade remains unknown. Plastid genomes of *C. callicephala* and *C. purpurea* showed high similarity, although some differences do exist. Whether the observed differences in the lengths of the single-copy and inverted-repeat regions and in the positions of their boundaries consistently distinguish these taxa remains to be tested across populations.

## Figures and Tables

**Table 1:** Lengths of the *rbcL*–*psaI* fragments obtained by *in silico* and *in vitro* amplification with primers rbcL_01_Fwd and psaI_01_Rev. The *Lactuca sativa* value is an *in silico* prediction.

Species	Expected Fragment Size (bp)	Observed on Gel
*Crepis callicephala*	1234	∼1.2 kbp
*C. purpurea*	1228	∼1.2 kbp
*L. sativa*	3066	not tested

**Table 2:** Primers designed on the *Crepis callicephala* plastome, with their target fragments and expected product sizes.

Primer Label	Target Fragment	Sequence ($5^{\prime }\to 3^{\prime }$)	Tm (^∘^C)	Expected Product Size (bp)
rbcL_01_Fwd	*rbcL*–*psaI* region	GAACTTTAGGGCACCCTTGG	58.5	1234
psaI_01_Rev	*rbcL*–*psaI* region	GAAATAAAGAAGCCATTGCGA	58.5	1234
IRA_LSC_Fw	IRa/LSC junction (JLA)	CCGGATCTAAGCGTTGGCT	60.0	564
IRA_LSC_Rv	IRa/LSC junction (JLA)	TCCAATGGAATTTCGCTCTAAAATA	60.0	564
LSC_IRB_Fw	LSC/IRb junction (JLB)	TTATCGCTTTTTGCATGTCCTCG	60.0	530
IRB_SSC_Fw	IRb/SSC junction (JSB)	TTCTCCGAGCTCACGTTATGG	60.0	584
IRB_SSC_Rv	IRb/SSC junction (JSB)	TCGGGGTTACATAGATGCCT	60.0	584
SSC_IRA_Fw	SSC/IRa junction (JSA)	TACTCAAAATGGGTGACGGC	60.0	609

For the LSC/IRb (JLB) and SSC/IRa (JSA) junctions, a single primer was paired with one from the other repeat copy—LSC_IRB_Fw with IRA_LSC_Fw, and SSC_IRA_Fw with IRB_SSC_Fw—which anneals in the opposite orientation because the two inverted repeats are reverse-complementary.

## Data Availability

The plastid genome sequences are available in GenBank as PZ258326.1 (material identified in this study as *C. callicephala*) and PZ336829.1 (material identified as *C. purpurea*). Both accessions are indexed under *C. purpurea* in NCBI Taxonomy, where *C. callicephala* is currently listed as a heterotypic synonym (TaxID 509683). Processed plastome sequences are available in the Zenodo repository (https://doi.org/10.5281/zenodo.10989900).

## Supplementary Materials

The following supporting information can be downloaded at https://www.mdpi.com/article/10.3390/ijms27219446/s1.
