# Supplementary material for: Phylogenetic Analysis of Rare Crepis Species of the Crimean Flora (C. purpurea (Willd.) M. Bieb. and C. callicephala Juz.) Through Plastid Genome Reconstruction
Source: Int J Mol Sci. 2026 Oct 23;27(21):9446. doi: 10.3390/ijms27219446 (PMC13762634; doi:10.3390/ijms27219446)
Supplement: Supplementary file 1 [file ijms-27-09446-s001.zip › Figure_S4_Crepidinae_ML_tree_RAxML-NG.pdf]

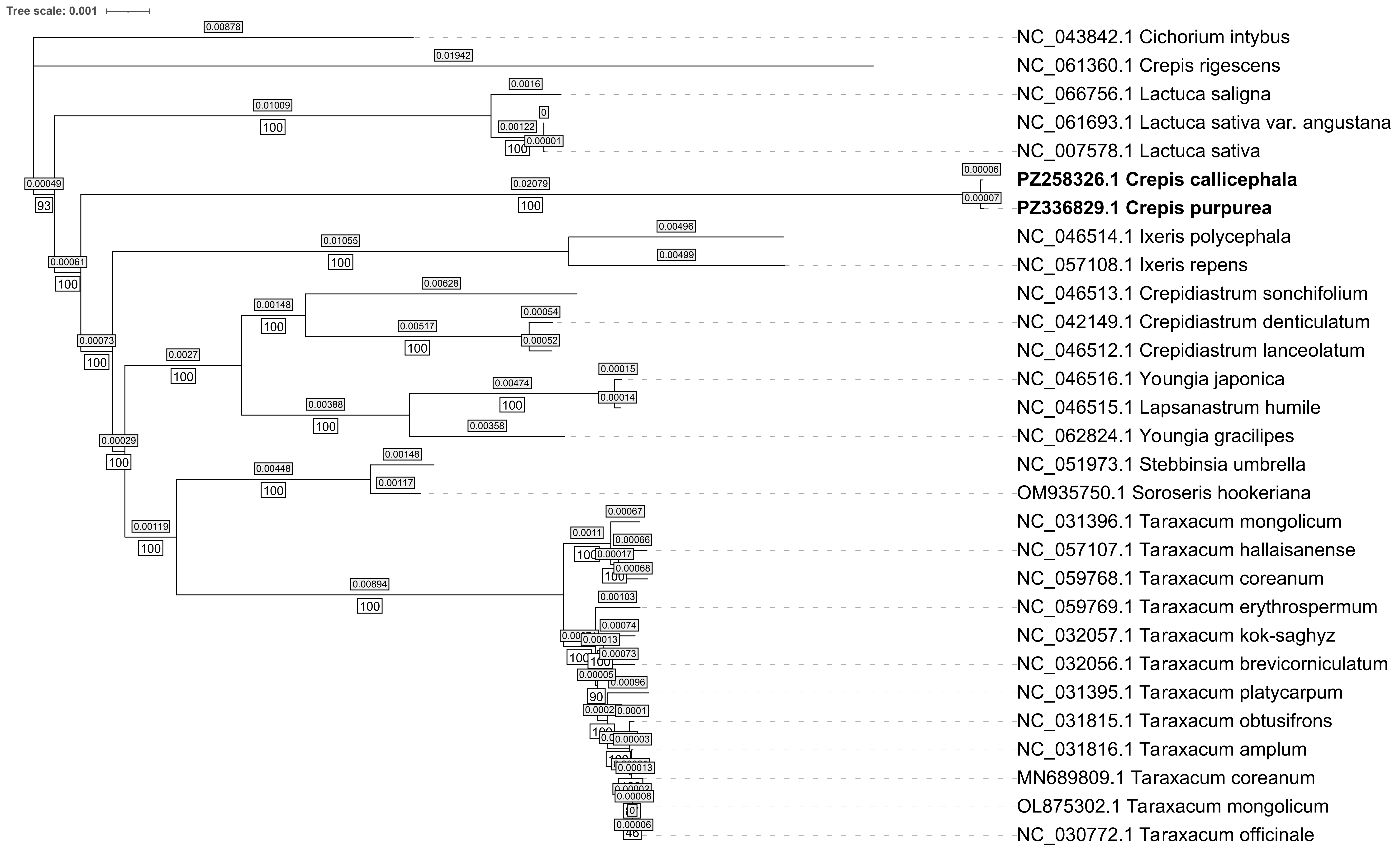

Tree scale: 0.001
NC_043842.1 Cichorium intybus
NC_061360.1 Crepis rigescens
NC_066756.1 Lactuca saligna
NC_061693.1 Lactuca sativa var. angustana
NC_007578.1 Lactuca sativa
PZ258326.1 Crepis callicephala
PZ336829.1 Crepis purpurea
NC_046514.1 Ixeris polycephala
NC_057108.1 Ixeris repens
NC_046513.1 Crepidiastrum sonchifolium
NC_042149.1 Crepidiastrum denticulatum
NC_046512.1 Crepidiastrum lanceolatum
NC_046516.1 Youngia japonica
NC_046515.1 Lapsanastrum humile
NC_062824.1 Youngia gracilipes
NC_051973.1 Stebbinsia umbrella
OM935750.1 Soroseris hookeriana
NC_031396.1 Taraxacum mongolicum
NC_057107.1 Taraxacum hallaisanense
NC_059768.1 Taraxacum coreanum
NC_059769.1 Taraxacum erythrospermum
NC_032057.1 Taraxacum kok-saghyz
NC_032056.1 Taraxacum brevicorniculatum
NC_031395.1 Taraxacum platycarpum
NC_031815.1 Taraxacum obtusifrons
NC_031816.1 Taraxacum amplum
MN689809.1 Taraxacum coreanum
OL875302.1 Taraxacum mongolicum
NC_030772.1 Taraxacum officinale
