# Supplementary material for: Phylogenetic Analysis of Rare Crepis Species of the Crimean Flora (C. purpurea (Willd.) M. Bieb. and C. callicephala Juz.) Through Plastid Genome Reconstruction
Source: Int J Mol Sci. 2026 Oct 23;27(21):9446. doi: 10.3390/ijms27219446 (PMC13762634; doi:10.3390/ijms27219446)
Supplement: Supplementary file 1 [file ijms-27-09446-s001.zip › Figure_S5_Crepidinae_ML_tree_IQ-TREE.pdf]

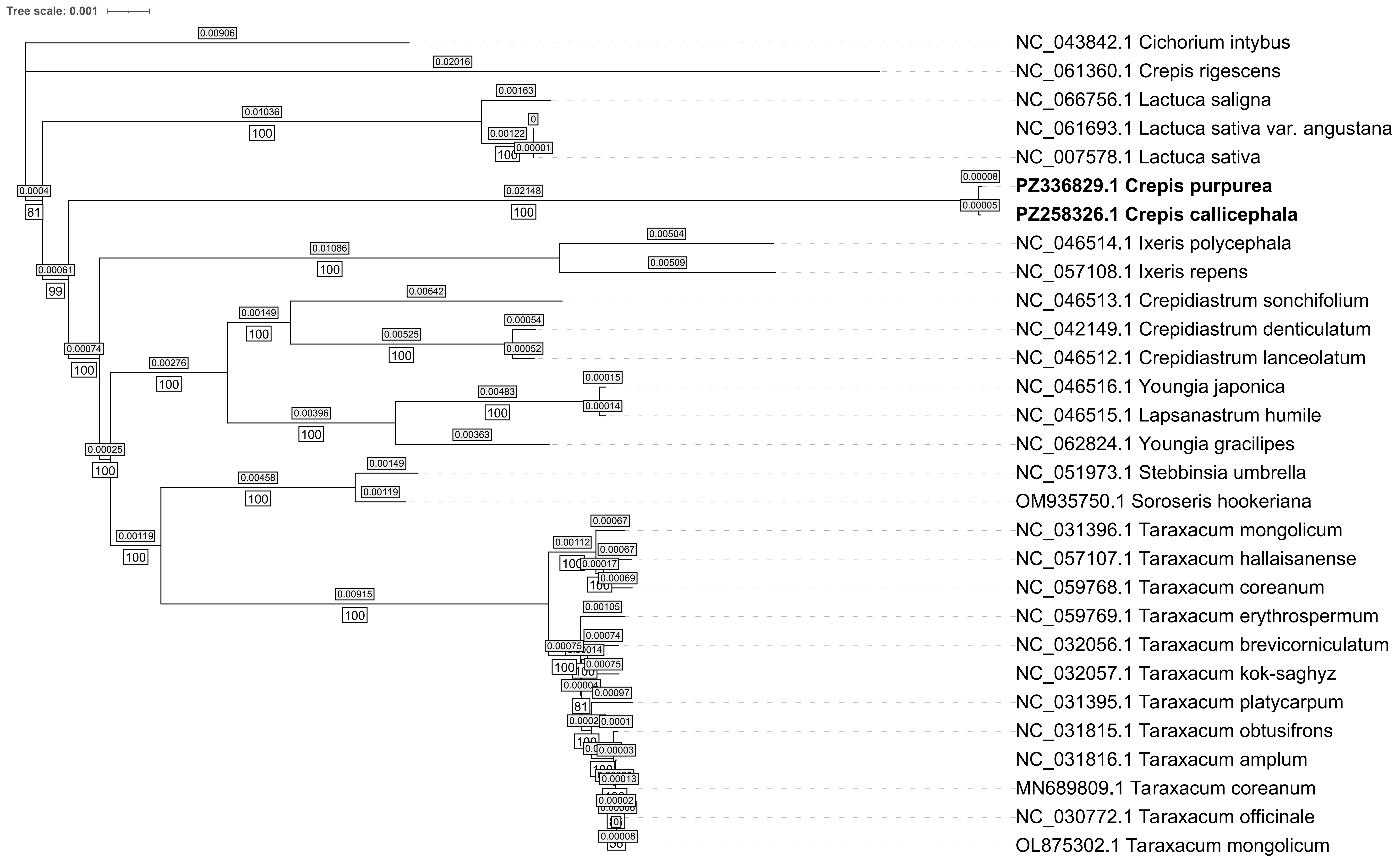
Tree scale: 0.001
NC_043842.1 Cichorium intybus
NC_061360.1 Crepis rigescens
NC_066756.1 Lactuca saligna
NC_061693.1 Lactuca sativa var. angustana
NC_007578.1 Lactuca sativa
PZ336829.1 Crepis purpurea
PZ258326.1 Crepis callicephala
NC_046514.1 Ixeris polycephala
NC_057108.1 Ixeris repens
NC_046513.1 Crepidiastrum sonchifolium
NC_042149.1 Crepidiastrum denticulatum
NC_046512.1 Crepidiastrum lanceolatum
NC_046516.1 Youngia japonica
NC_046515.1 Lapsanastrum humile
NC_062824.1 Youngia gracilipes
NC_051973.1 Stebbinsia umbrella
OM935750.1 Soroseris hookeriana
NC_031396.1 Taraxacum mongolicum
NC_057107.1 Taraxacum hallaisanense
NC_059768.1 Taraxacum coreanum
NC_059769.1 Taraxacum erythrospermum
NC_032056.1 Taraxacum brevicorniculatum
NC_032057.1 Taraxacum kok-saghyz
NC_031395.1 Taraxacum platycarpum
NC_031815.1 Taraxacum obtusifrons
NC_031816.1 Taraxacum amplum
MN689809.1 Taraxacum coreanum
NC_030772.1 Taraxacum officinale
OL875302.1 Taraxacum mongolicum
0.00906
0.02016
0.00163
0.01036
100
0
0.00122
0.00001
100
0.0004
81
0.02148
100
0.00008
0.00005
0.00504
0.01086
100
0.00509
0.00061
99
0.00642
0.00149
100
0.00054
0.00525
100
0.00052
0.00074
100
0.00276
100
0.00015
0.00483
100
0.00014
0.00396
100
0.00363
0.00025
100
0.00149
0.00458
100
0.00119
0.00119
100
0.00067
0.00112
0.00067
0.00017
0.00069
0.00915
100
0.00105
0.00074
0.00075
100
0.00075
0.00097
81
0.0001
0.00003
0.00013
0.00002
0.00008
