# Supplementary material for: Phylogenetic Analysis of Rare Crepis Species of the Crimean Flora (C. purpurea (Willd.) M. Bieb. and C. callicephala Juz.) Through Plastid Genome Reconstruction
Source: Int J Mol Sci. 2026 Oct 23;27(21):9446. doi: 10.3390/ijms27219446 (PMC13762634; doi:10.3390/ijms27219446)
Supplement: Supplementary file 1 [file ijms-27-09446-s001.zip › Table_S1_QUAST_assemblies.pdf]

Report

	pomoxis
# contigs (>= 0 bp)	1
# contigs (>= 1000 bp)	1
# contigs (>= 5000 bp)	1
# contigs (>= 10000 bp)	1
# contigs (>= 25000 bp)	1
# contigs (>= 50000 bp)	1
Total length (>= 0 bp)	149790
Total length (>= 1000 bp)	149790
Total length (>= 5000 bp)	149790
Total length (>= 10000 bp)	149790
Total length (>= 25000 bp)	149790
Total length (>= 50000 bp)	149790
# contigs	1
Largest contig	149790
Total length	149790
Reference length	149980
GC (%)	37.70
Reference GC (%)	37.70
N50	149790
NG50	149790
N90	149790
NG90	149790
auN	149790.0
auNG	149600.2
L50	1
LG50	1
L90	1
LG90	1
# misassemblies	0
# misassembled contigs	0
Misassembled contigs length	0
# local misassemblies	0
# scaffold gap ext. mis.	0
# scaffold gap loc. mis.	0
# unaligned mis. contigs	0
# unaligned contigs	0 + 0 part
Unaligned length	0
Genome fraction (%)	99.878
Duplication ratio	1.000
# N's per 100 kbp	0.00
# mismatches per 100 kbp	2.67
# indels per 100 kbp	16.69
# genomic features	366 + 5 part
Largest alignment	149790
Total aligned length	149790
NA50	149790
NGA50	149790
NA90	149790
NGA90	149790
auNA	149790.0
auNGA	149600.2
LA50	1
LGA50	1
LA90	1
LGA90	1

All statistics are based on contigs of size >= 500 bp, unless otherwise noted (e.g., "# contigs (>= 0 bp)" and "Total length (>= 0 bp)" include all contigs).

Misassemblies report

	pomoxis
# misassemblies	0
# contig misassemblies	0
# c. relocations	0
# c. translocations	0
# c. inversions	0
# scaffold misassemblies	0
# s. relocations	0
# s. translocations	0
# s. inversions	0
# misassembled contigs	0
Misassembled contigs length	0
# local misassemblies	0
# scaffold gap ext. mis.	0
# scaffold gap loc. mis.	0
# unaligned mis. contigs	0
# mismatches	4
# indels	25
# indels (<= 5 bp)	25
# indels (> 5 bp)	0
Indels length	33

All statistics are based on contigs of size >= 500 bp, unless otherwise noted (e.g., "# contigs (>= 0 bp)" and "Total length (>= 0 bp)" include all contigs).

Unaligned report

	pomoxis
# fully unaligned contigs	0
Fully unaligned length	0
# partially unaligned contigs	0
Partially unaligned length	0
# N's	0

All statistics are based on contigs of size >= 500 bp, unless otherwise noted (e.g., "# contigs (>= 0 bp)" and "Total length (>= 0 bp)" include all contigs).

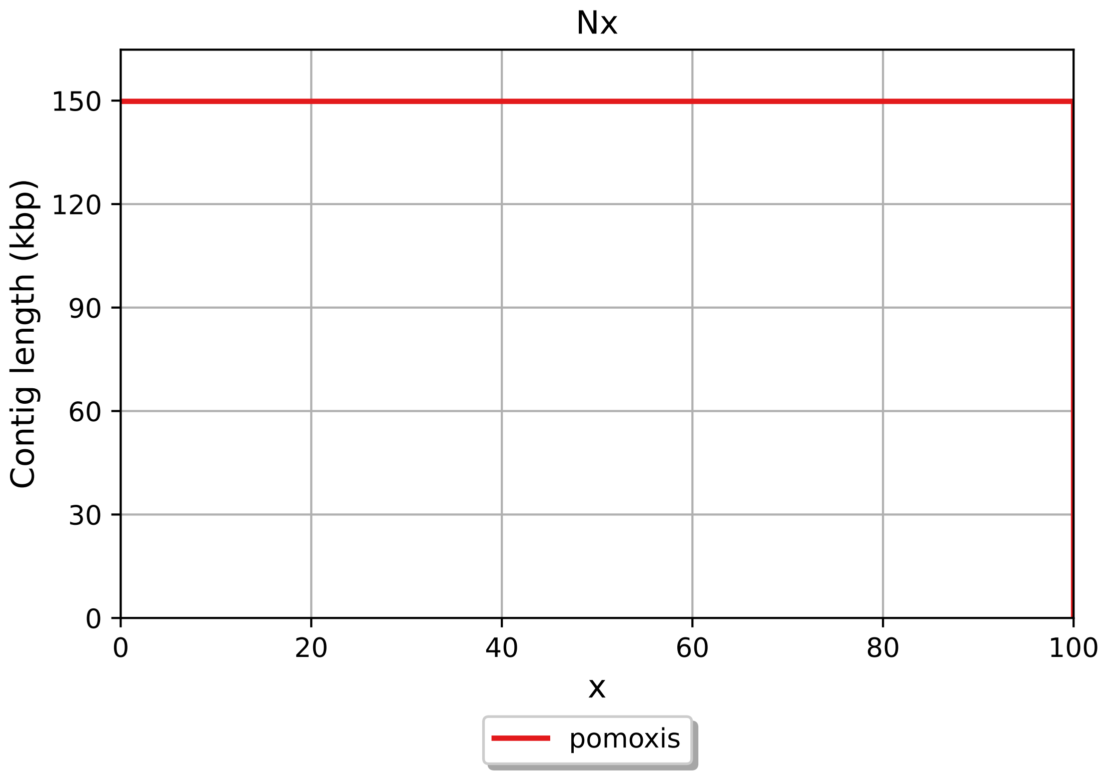

Nx
Contig length (kbp)
150
120
90
60
30
0
0
20
40
60
80
100
x
pomoxis

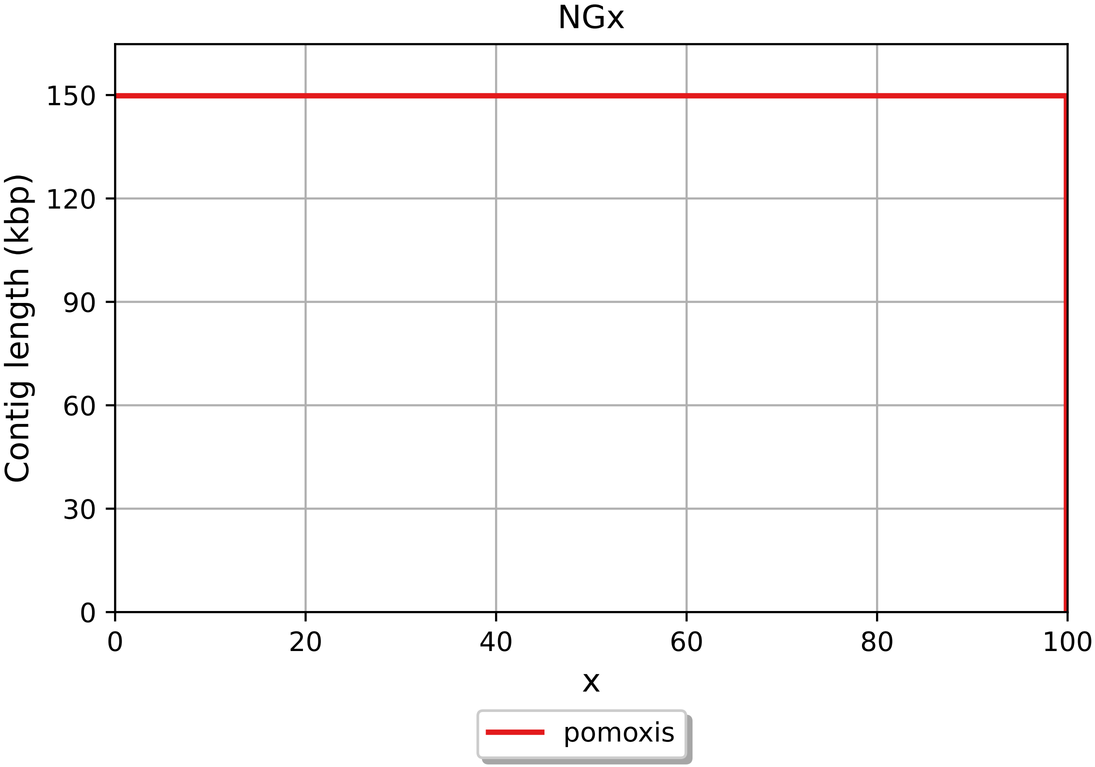
NGx
Contig length (kbp)
150
120
90
60
30
0
0
20
40
60
80
100
x
pomoxis

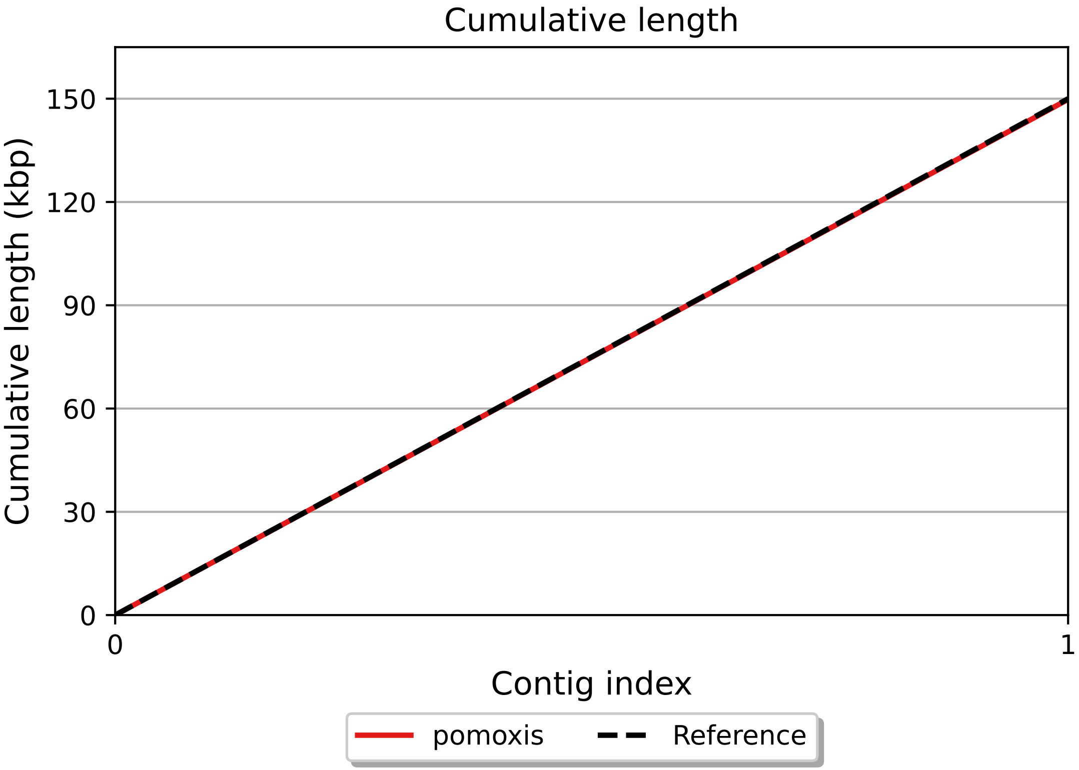

Cumulative length
Cumulative length (kbp)
0
30
60
90
120
150
0
1
Contig index
pomoxis
Reference

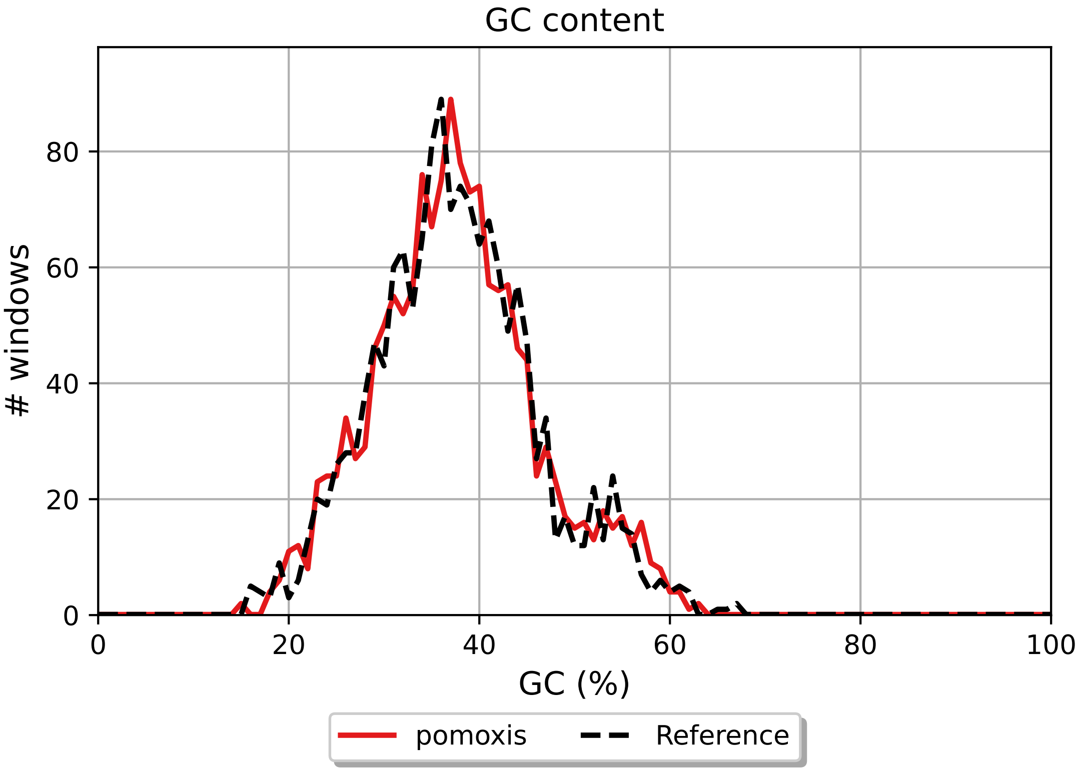
GC content
windows
GC (%)
0
20
40
60
80
100
pomoxis
Reference

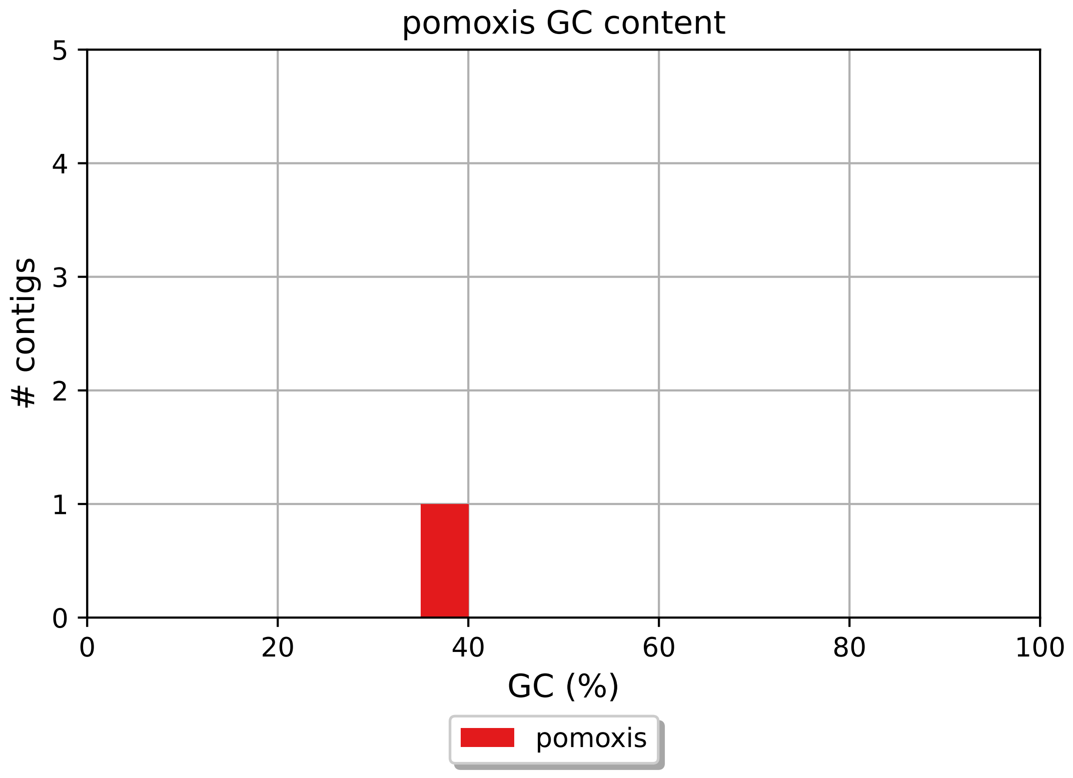
pomoxis GC content
contigs
GC (%)
0
1
2
3
4
5
0
20
40
60
80
100
pomoxis

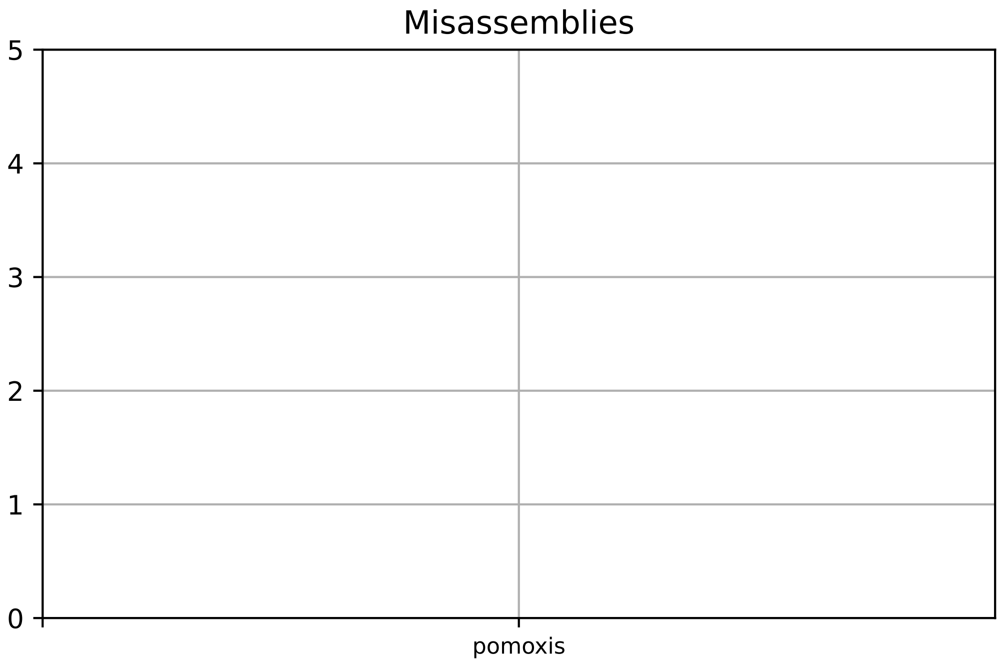
Misassemblies
5
4
3
2
1
0
pomoxis

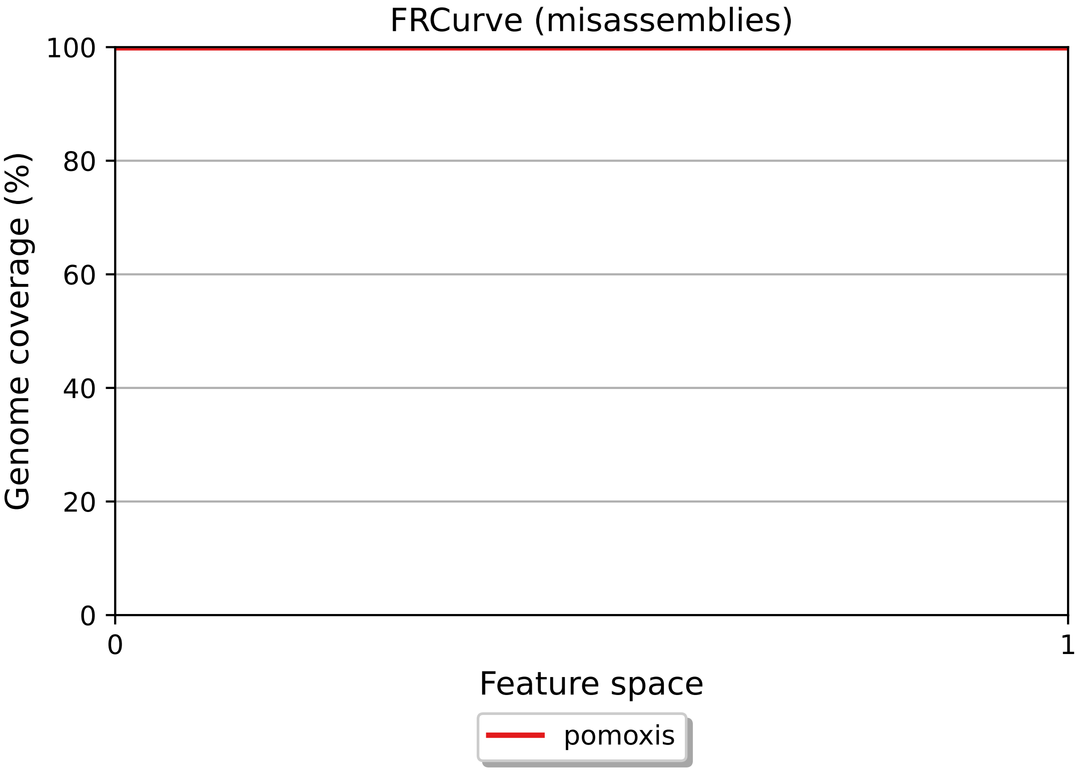
FRCurve (misassemblies)
Genome coverage (%)
100
80
60
40
20
0
0
1
Feature space
pomoxis

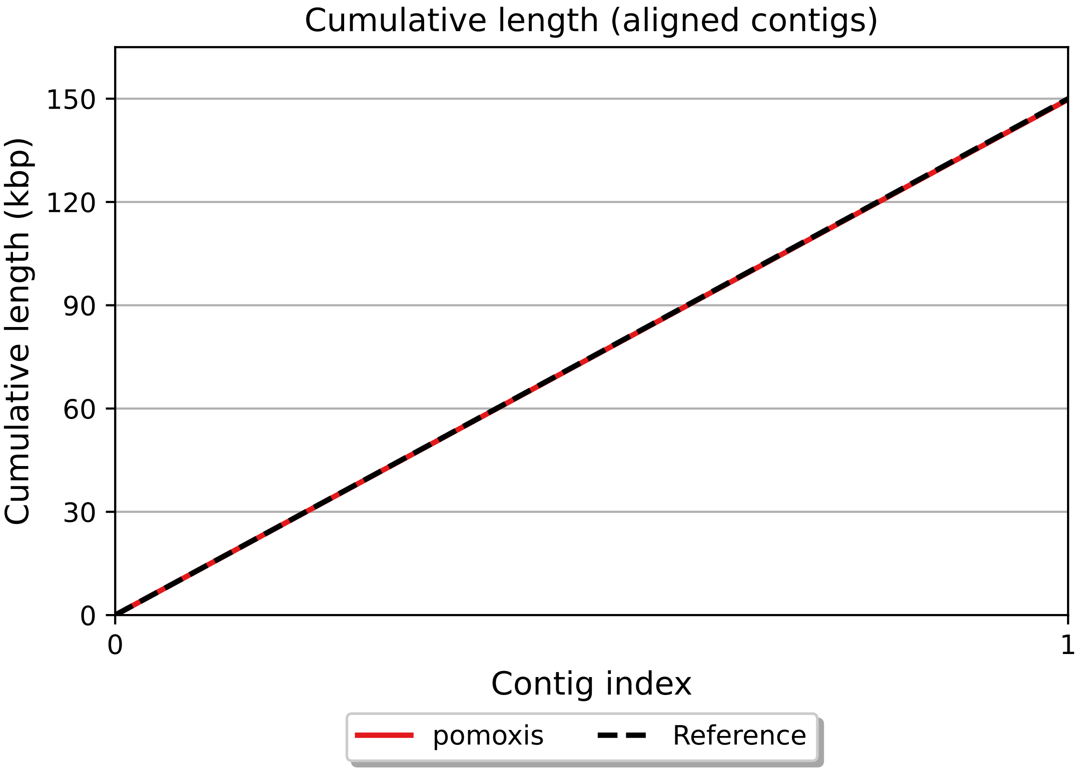
Cumulative length (aligned contigs)
Cumulative length (kbp)
150
120
90
60
30
0
0
1
Contig index
pomoxis
Reference

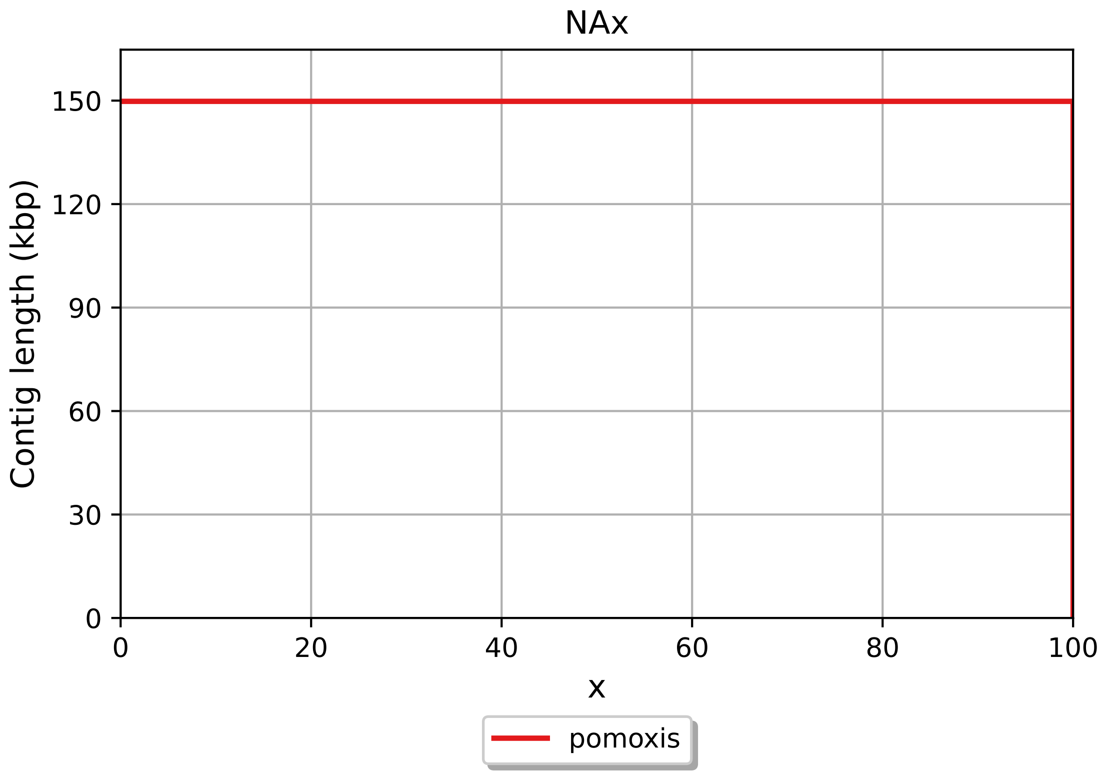
NAx
Contig length (kbp)
150
120
90
60
30
0
0
20
40
60
80
100
x
pomoxis

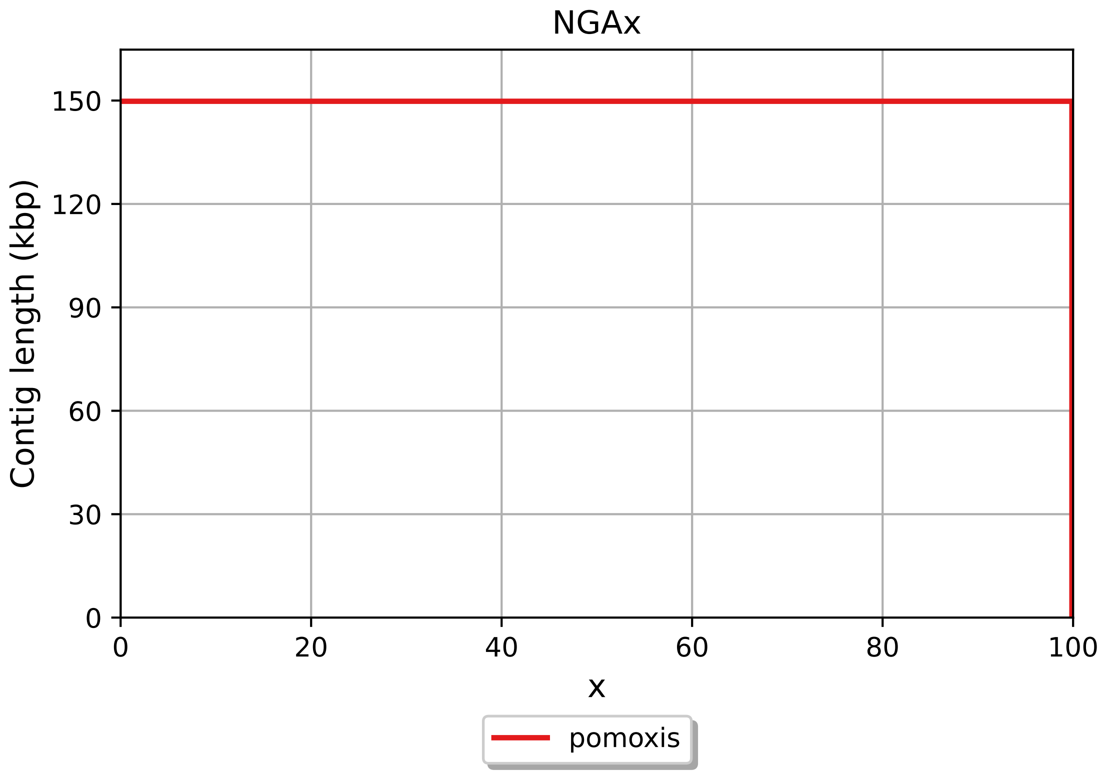
NGAx
Contig length (kbp)
150
120
90
60
30
0
0
20
40
60
80
100
x
pomoxis

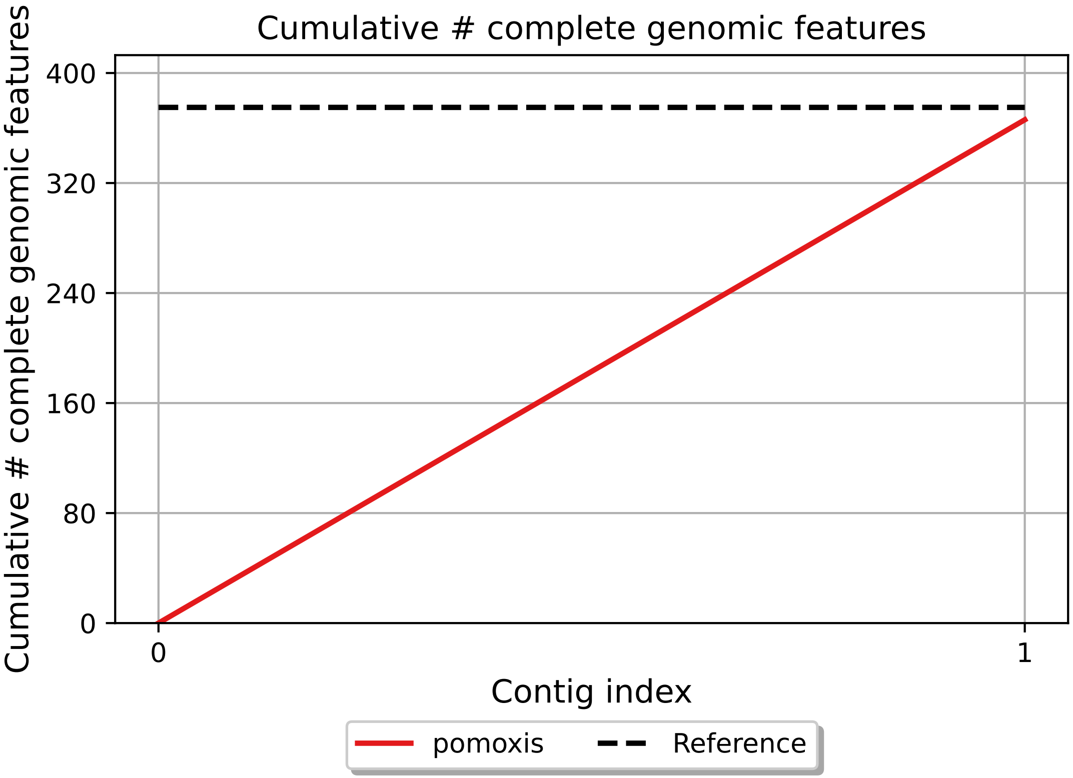
Cumulative # complete genomic features
Cumulative # complete genomic features
400
320
240
160
80
0
0
1
Contig index
pomoxis
Reference

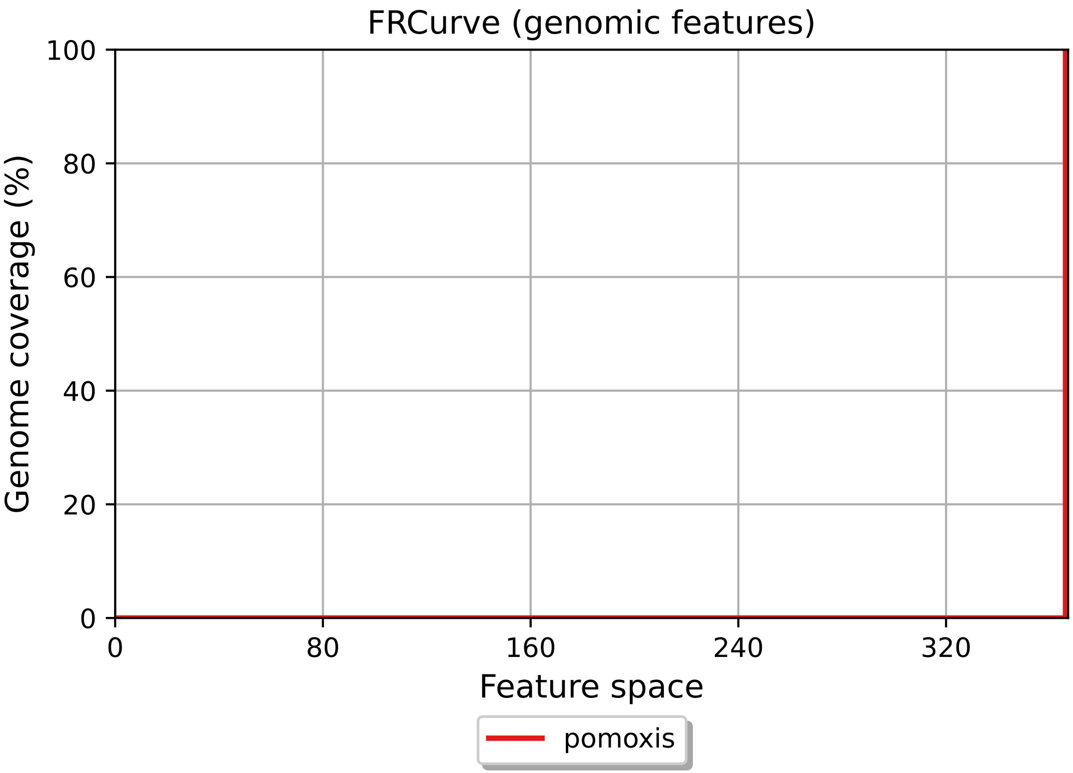
FRCurve (genomic features)
Genome coverage (%)
0
20
40
60
80
100
0
80
160
240
320
Feature space
pomoxis
